# A new TROP2-targeting antibody-drug conjugate shows potent antitumor efficacy in breast and lung cancers

**DOI:** 10.1038/s41698-024-00584-z

**Published:** 2024-04-23

**Authors:** Dan-dan Zhou, Xiao-tian Zhai, Lan-wen Zhang, Zi-hui Xie, Ying Wang, Yong-su Zhen, Rui-juan Gao, Qing-fang Miao

**Affiliations:** NHC Key Laboratory of Biotechnology for Microbial Drugs, Institute of Medicinal Biotechnology, Chinese Academy of Medical Sciences & Peking Union Medical College, Beijing, China

**Keywords:** Targeted therapies, Breast cancer, Lung cancer

## Abstract

Trophoblast cell surface antigen 2 (Trop2) is considered to be an attractive therapeutic target in cancer treatments. We previously generated a new humanized anti-Trop2 antibody named hIMB1636, and designated it as an ideal targeting carrier for cancer therapy. Lidamycin (LDM) is a new antitumor antibiotic, containing an active enediyne chromophore (AE) and a noncovalently bound apoprotein (LDP). AE and LDP can be separated and reassembled, and the reassembled LDM possesses cytotoxicity similar to that of native LDM; this has made LDM attractive in the preparation of gene-engineering drugs. We herein firstly prepared a new fusion protein hIMB1636-LDP composed of hIMB1636 and LDP by genetic engineering. This construct showed potent binding activities to recombinant antigen with a K_D_ value of 4.57 nM, exhibited binding to Trop2-positive cancer cells and internalization and transport to lysosomes, and demonstrated powerful tumor-targeting ability in vivo. We then obtained the antibody-drug conjugate (ADC) hIMB1636-LDP-AE by molecular reconstitution. In vitro, hIMB1636-LDP-AE inhibited the proliferation, migration, and tumorsphere formation of tumor cells with half-maximal inhibitory concentration (IC_50_) values at the sub-nanomolar level. Mechanistically, hIMB1636-LDP-AE induced apoptosis and cell-cycle arrest. In vivo, hIMB1636-LDP-AE also inhibited the growth of breast and lung cancers in xenograft models. Moreover, compared to sacituzumab govitecan, hIMB1636-LDP-AE showed more potent antitumor activity and significantly lower myelotoxicity in tumors with moderate Trop2 expression. This study fully revealed the potent antitumor efficacy of hIMB1636-LDP-AE, and also provided a new preparation method for LDM-based ADC, as well as a promising candidate for breast cancer and lung cancer therapeutics.

## Introduction

As a major public health issue, cancer is the second leading cause of death worldwide^1^. Based on statistics from the GLOBOCAN^2^, the number of cancer cases is growing rapidly worldwide, and in 2020, the number of cases increased to 19.3 million, with nearly 10 million people having died from cancer. Therein, breast cancer is the most common cancer in the USA and lung cancer in China^3^, and lung cancer also remained the leading cause of cancer death, with 18% of total cancer deaths^2^. Breast cancer survival has improved significantly as a result of hormone therapy, chemotherapy, and radiotherapy. However, the incidence rate of breast cancer has remained high, with up to 24.5% of tumor cases in women^2^. Breast cancer is still a primary cause of cancer-related death in women, which profoundly reflects its heterogeneity, metastasis, and treatment resistance^4^. Over the years, the treatment of lung cancer has also evolved with the introduction of several lines of tyrosine kinase inhibitors in patients with EGFR, ALK, ROS1, and NTRK mutations. Similarly, immune checkpoint inhibitors (ICIs) have dramatically changed the landscape of lung cancer treatment. Unfortunately, lung cancer continues to have one of the worst 5-year survival rates among all cancer types^5^. Despite the ongoing development of new treatment regimens, there is an unmet clinical need in lung and breast cancer; thus, developing promising pharmacological strategies to improve patients’ clinical outcomes in both of these cancers is crucial.

Antibody-drug conjugates (ADC), known as “biological missiles”, are an emerging and rapidly developing class of targeted therapeutic agents^6^. As the name implies, it consists of a tumor-targeting antibody conjugated with a cytotoxic payload through a sophisticatedly designed chemical linker, enabling simultaneous selective targeting and potent toxicity. In 2000, the U.S. Food and Drug Administration (FDA) first approved the ADC drug, Mylotarg® (gemtuzumab ozogamicin), for adults with acute myeloid leukemia (AML), which marked the beginning of the ADC era of cancer-targeted therapy^7^. ADC development was on the rise evidenced by the approval of Adcetris® (brentuximab vedotin) in 2011 for the treatment of CD30-positive lymphomas and Kadcyla® (ado-trastuzumab emtansine) in 2013 for the treatment of HER2-positive breast cancer^8,9^. So far, 15 ADCs have received market approval worldwide, for targeted treatments of hematologic malignancies and solid tumors^10^. The key requirements for a successful ADC include selection of an appropriate target, antibody, linker, and cytotoxic payloads, all of which affect its druggability characteristics such as antitumor effects, pharmacokinetics, stability, and cytotoxicity^11,12^. Therefore, although the concept of an ADC is clear and straightforward, it is still challenging to develop an ideal ADC with the appropriate combination of antibody, linker, and payload, and as a result, commercially available ADCs are still limited.

Choosing a suitable target is the primary consideration for the design of a new ADC. Trophoblast cell surface antigen 2 (Trop2), encoded by *TACSTD2*, also known as tumor-associated calcium signal transducer 2, is a 36-kDa cell surface glycoprotein. Trop2 is upregulated in a variety of malignant tumors and participates in several oncogenic signaling pathways that lead to tumor development, invasion, and metastasis, but exhibits limited expression in normal human tissues^13^. Therefore, Trop2 is considered an attractive therapeutic target in cancer treatment, especially in the development of an ADC. One of the most successful cases for Trop2-targeted therapy is sacituzumab govitecan (SG; IMMU-132), which contains a humanized anti-Trop-2 monoclonal antibody and the topoisomerase I inhibitor drug SN-38. It has been approved by the U.S. Food and Drug Administration (FDA) for the treatment of patients with metastatic triple-negative breast cancer^14^.

The cytotoxic molecules used as payloads of ADCs should be highly effective, usually with half-maximal inhibitory concentration (IC_50_) in the nanomolar or picomolar range^12^. Lidamycin (LDM), derived from *Streptomyces globisporus* C1027, also called C-1027, is a member of the enediyne antibiotic family. It has shown extremely potent cytotoxicity and distinct antitumor effects in various types of cancer, including liver, breast, pancreatic, colon, lung, gastric, and brain cancer^15–17^. LDM contains an active enediyne chromophore (AE) responsible for extremely potent bioactivity and a noncovalently bound apoprotein (LDP), which forms a hydrophobic pocket for protecting the chromophore. AE and LDP can be separated and reassembled freely, and the reassembled LDM exhibits similar cytotoxicity to native LDM. LDM has been applied in clinical trials for cancer therapy, but its clinical progress has not been smooth due to its narrow therapeutic window and toxicity issues. Nonetheless, the characteristics of its structure and powerful cytotoxicity as well as antitumor effects have resulted in LDM attracting more attention in the development of gene engineering drugs and immunoconjugates.

In a previous study, we obtained the humanized anti-Trop2 antibody, hIMB1636, by traditional hybridoma technology and humanization via complementarity-determining region (CDR) grafting^18^. When hIMB1636 was radiolabeled with ^64^Cu/^177^Lu via p-SCN-Bn-NOTA (NOTA)/DOTA-NHS-ester (DOTA), ^64^Cu/^177^Lu-hIMB1636 demonstrated excellent imaging effects and significant antitumor efficacy in cell-derived tumor models of pancreatic cancer^19^, suggesting that hIMB1636 could be a targeting carrier for cancer therapy. Additionally, we also found that hIMB1636 could be internalized and trafficked to lysosomes by Trop2-positive cancer cells. In this study, according to the molecular properties of LDM, we first prepared a new ADC (hIMB1636-LDP-AE) composed of hIMB1636 and LDM by the combination of genetic engineering and molecular reconstitution and then studied its activities against breast cancer and lung cancer both in vitro and in vivo. This study fully demonstrated the potential efficacy of hIMB1636-LDP-AE against solid tumors, which not only provides a new preparation method for LDM-based ADCs but also provides a promising candidate for the treatment of breast cancer and lung cancer.

## Results

### Generation and characterization of hIMB1636-LDP protein

**Fig. 1:**
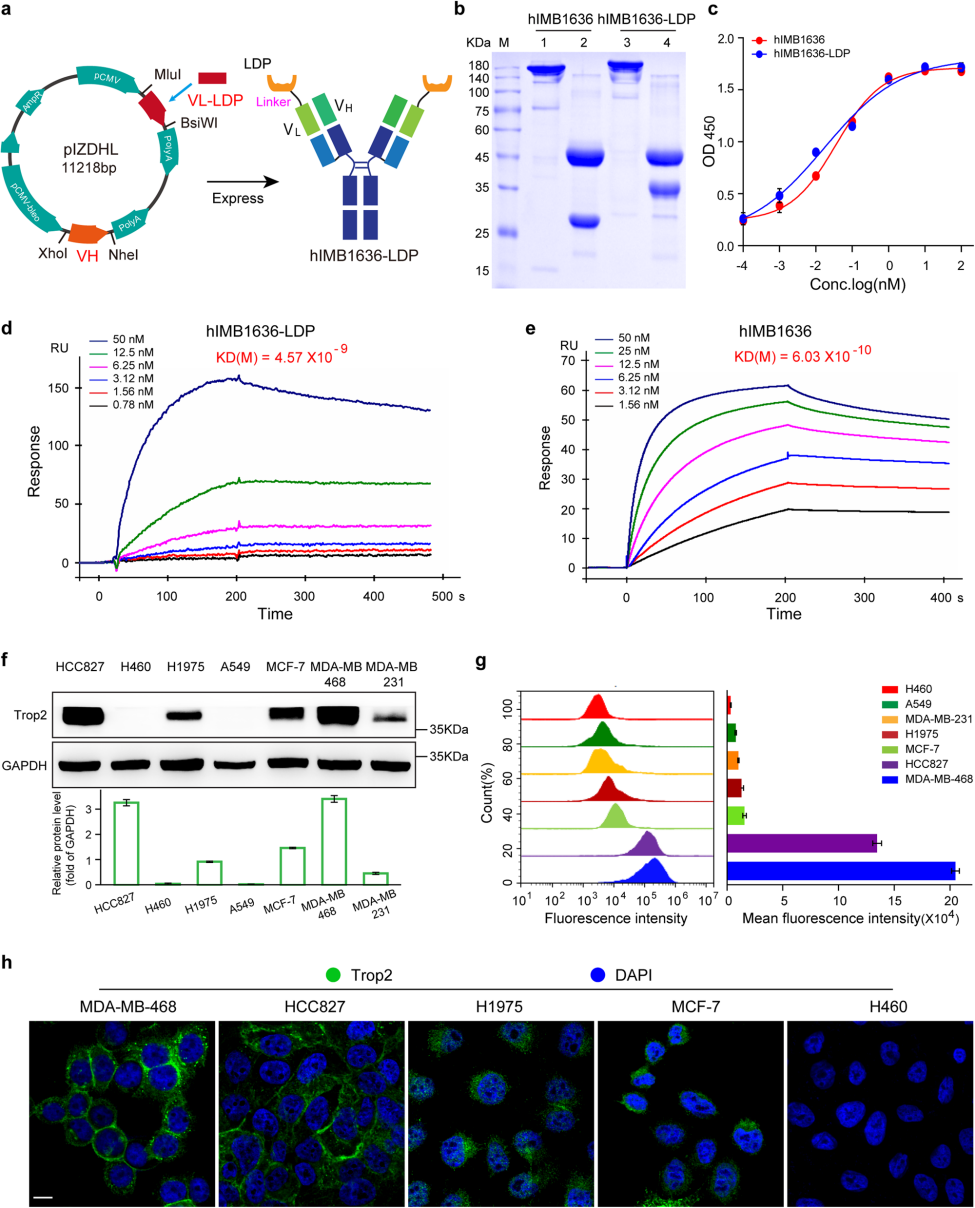
Characterization of hIMB1636-LDP protein in vitro. **a** Construction diagram of the hIMB1636-LDP fusion protein. **b** SDS-PAGE analysis of hIMB1636-LDP and hIMB1636 antibody under reducing and non-reducing conditions. **c** Binding activity of hIMB1636-LDP and hIMB1636 to recombinant human Trop2 antigen by ELISA. Data represent mean ± SD, *n* = 3. **d**, **e** SPR analysis of hIMB1636-LDP (**d**) or hIMB1636 antibody (**e**) binding with Trop2 antigen. **f** Detection of Trop2 protein expression in different cancer cells by western-blot. Data represent mean ± SD, *n* = 3. **g** Binding activity of hIMB1636-LDP antibody to different tumor cell lines by flow-cytometric analysis. The horizontal axis represents the values of MFI (mean fluorescence intensity) values. Data represent mean ± SD, *n* = 3. **h** Binding activity of hIMB1636-LDP antibody to cancer cells by immunofluorescence assay.

To generate a new ADC (referred to herein as hIMB1636-LDP-AE) consisting of a humanized antibody hIMB1636 directed against Trop2 and the potent cytotoxic agent LDM, we first constructed an expression vector of hIMB1636-LDP protein by genetic engineering. In brief, the LDP sequence of LDM was linked to the N-terminal of VL of the hIMB1636 light chain by a non-cleavable peptide SGGPEGGS and inserted into the expression vector pIZDHL (Fig. 1a). After transfection, screening, and purification, we obtained the hIMB1636-LDP fusion protein; hIMB1636 (naked anti-Trop2 antibody) was also expressed as a parallel control. SDS/PAGE analysis showed that under reducing conditions, the heavy-chain molecular weights (MWs) of hIMB1636 and hIMB1636-LDP were identical at 50 kDa, while the MWs of the light chains were approximately 25 and 35 kDa, respectively (Fig. 1b). The latter difference corresponded to the theoretical molecular weight for LDP of about 10 kDa, indicating that hIMB1636-LDP was correctly expressed. The antigen-binding properties of hIMB1636-LDP were crucial for its in vitro and in vivo activity. As shown in Fig. 1c, the saturation dose-response curve for hIMB1636-LDP and Trop2 antigen was consistent with that of parental hIMB1636 antibody and antigen. SPR results showed that hIMB1636-LDP reflected a potent binding affinity to Trop2 antigen, with an apparent equilibrium dissociation constant (K_D_) of 4.57 nM (Fig. 1d), slightly lower than that for naked hIMB1636 antibody (K_D_ = 0.603 nM) (Fig. 1e).

To mediate cytotoxicity, hIMB1636-LDP fusion protein must have the ability to bind the native antigen on cancer cells, and thus we first detected the expression levels of Trop2 in a variety of lung cancer and breast cancer cells. As shown in Fig. 1f, H1975, MCF-7, and MDA-MB-231 cells expressed moderate levels of Trop2; and there was elevated expression in HCC827 and MDA-MB-468 cells. In contrast, we determined virtually no Trop2 expression on H460 and A549 cells (Fig. 1f). Flow-cytometric assays revealed that the fluorescence signals for hIMB1636-LDP in H460, A549, MDA-MB-231, H1975, MCF-7, HCC827, and MDA-MB-468 cells increased commensurately (Fig. 1g), showing direct proportionality toward Trop2 expression via the western-blot results in Fig. 1f. Furthermore, immunofluorescence analysis showed that hIMB1636-LDP could bind to cells with high or moderate Trop2 expression at single cell level (Fig. 1h). These results indicated that hIMB1636-LDP bound to native antigen on the surface of cancer cells.

### Specific binding, endocytosis, and tumor targeting ability of hIMB1636-LDP

**Fig. 2:**
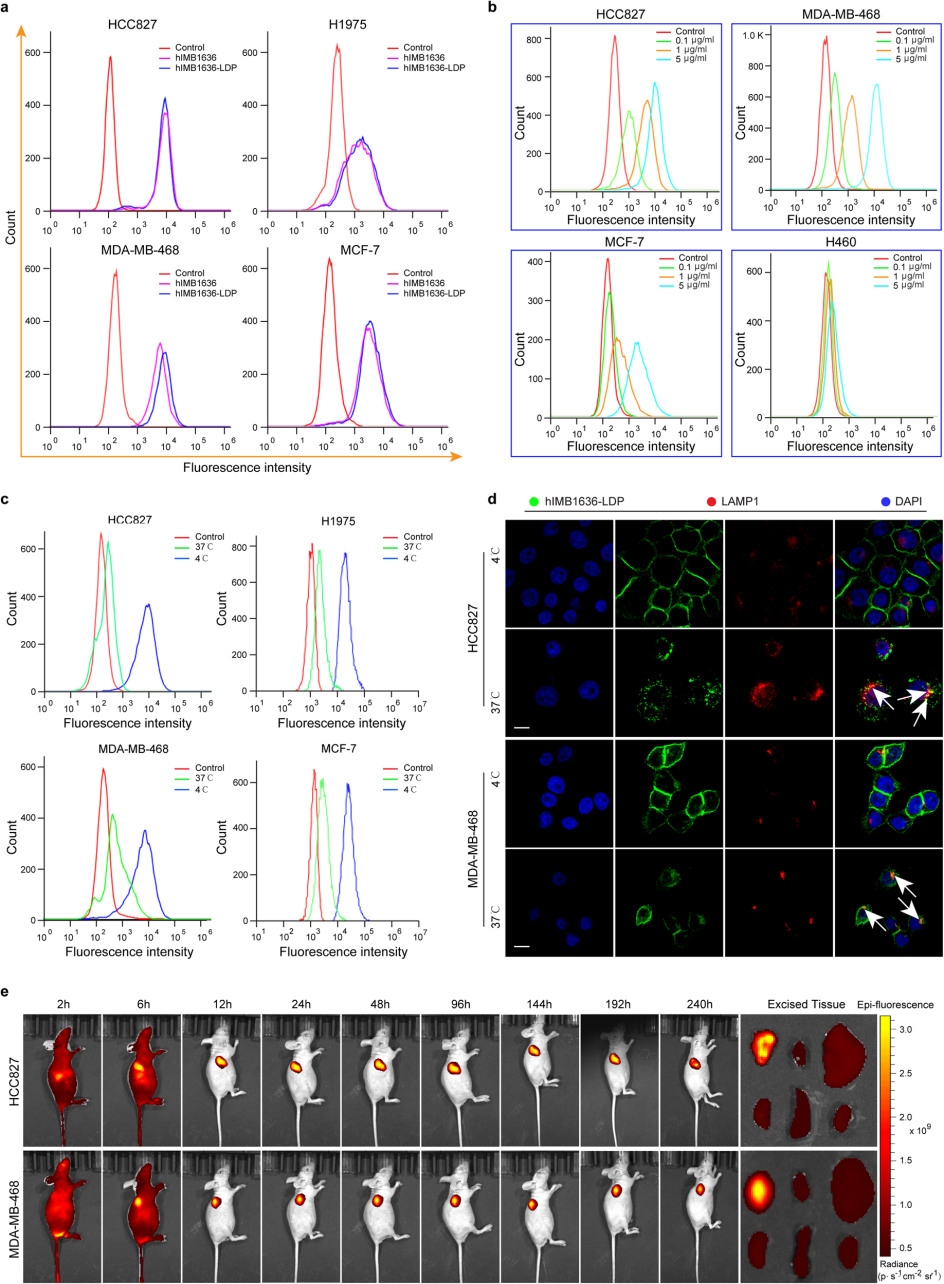
Binding specificity, internalization, and tumor-targeting capability of hIMB1636-LDP protein. **a** The binding activity of hIMB1636-LDP and hIMB1636 to native Trop2 antigen on tumor cells was analyzed by FCM. **b** Binding activities of hIMB1636-LDP at different concentrations to various cancer cell types by FCM analysis. **c** The binding of hIMB1636-LDP to Trop2-positive tumor cells at 37 °C and 4 °C were analyzed by FCM. **d** The internalization and lysosomal localization of hIMB1636-LDP in HCC827 and MDA-MB-468 cells by laser-scanning confocal microscopy. **e** Left: In vivo fluorescence imaging of hIMB1636-LDP with DyLight 680 labeling in HCC827 and MDA-MB-468 xenograft models. Right: Fluorescence images of tumorous and normal tissues (including heart, liver, lung, spleen, and kidney) in vitro. Color scale represents photons/s/cm^2^/steradian.

The binding affinities of hIMB1636-LDP and hIMB1636 antibodies to natural antigens were investigated by FCM. As shown in Fig. 2a, the fluorescence shifts were similar in HCC827 cells after being treated with 5 μg/mL hIMB1636-LDP or naked hIMB1636 antibody. The same results were obtained in H1975, MDA-MB-468, and MCF-7 cells, indicating that the linking of the LDP moiety did not induce any adverse effects on the binding activity of the parent antibody hIMB1636. We thereafter assessed the binding specificity of the hIMB1636-LDP antibody. As expected, the fluorescence shift for the binding of hIMB1636-LDP to high Trop2-expressing HCC827 and MDA-MB-468 cells was concentration-dependent and substantially larger than that in MCF-7 cells (which showed moderate Trop2 expression), while there was almost no shift for Trop2-negative H460 cells (Fig. 2b). Endocytosis is the key step in ADCs entering target cells, and we thus evaluated the internalization of hIMB1636-LDP in Trop2^+^ tumor cell lines. The fluorescence shift for hIMB1636-LDP binding to tumor cells at 37 °C was significantly less than at 4 °C (the latter temperature allows binding but not internalization^20^) (Fig. 2c), indirectly indicating that under normal physiologic conditions, hIMB1636-LDP was rapidly internalized into target cells after binding to antigens.

Immunofluorescence analysis directly confirmed that hIMB1636-LDP was internalized by cancer cells. As shown in Fig. 2d, hIMB1636-LDP staining (green fluorescence) was uniformly localized on the plasma membrane of HCC827 and MDA-MB-468 cells at 4 °C. However, after incubation at 37 °C, the green fluorescence signal on cell surfaces was decreased and scattered; and appeared in the lysosomes (red fluorescence), as evidenced by the presence of yellow fluorescence due to the overlap of staining for hIMB1636-LDP and the lysosomal marker LAMP-1 (Fig. 2d). These fluorescence signals indicated that hIMB1636-LDP was internalized and transported to the lysosomal compartment. The tumor-targeting ability of hIMB1636-LDP was subsequently evaluated using mouse xenograft models bearing the HCC827 or MDA-MB-468 cells, and the fluorescence signal was clearly visualized in the tumor sites within 6 h after i.v. administration of DyLight 680-labeled hIMB1636-LDP. In the HCC827 xenograft model, the fluorescence intensity continuously increased over time and reached a maximum of 48 h. The total tumor retention time of hIMB1636-LDP was over 240 h (Fig. 2e). Similarly, the fluorescence signal was also maintained for up to 10 days in the MDA-MB-468 xenograft model (Fig. 2e). More importantly—and except for tumor tissue—we noted no fluorescence signal in other organs such as heart, liver, lung, spleen, and kidney.

### Assembly of enediyne-integrated hIMB1636-LDP-AE

**Fig. 3:**
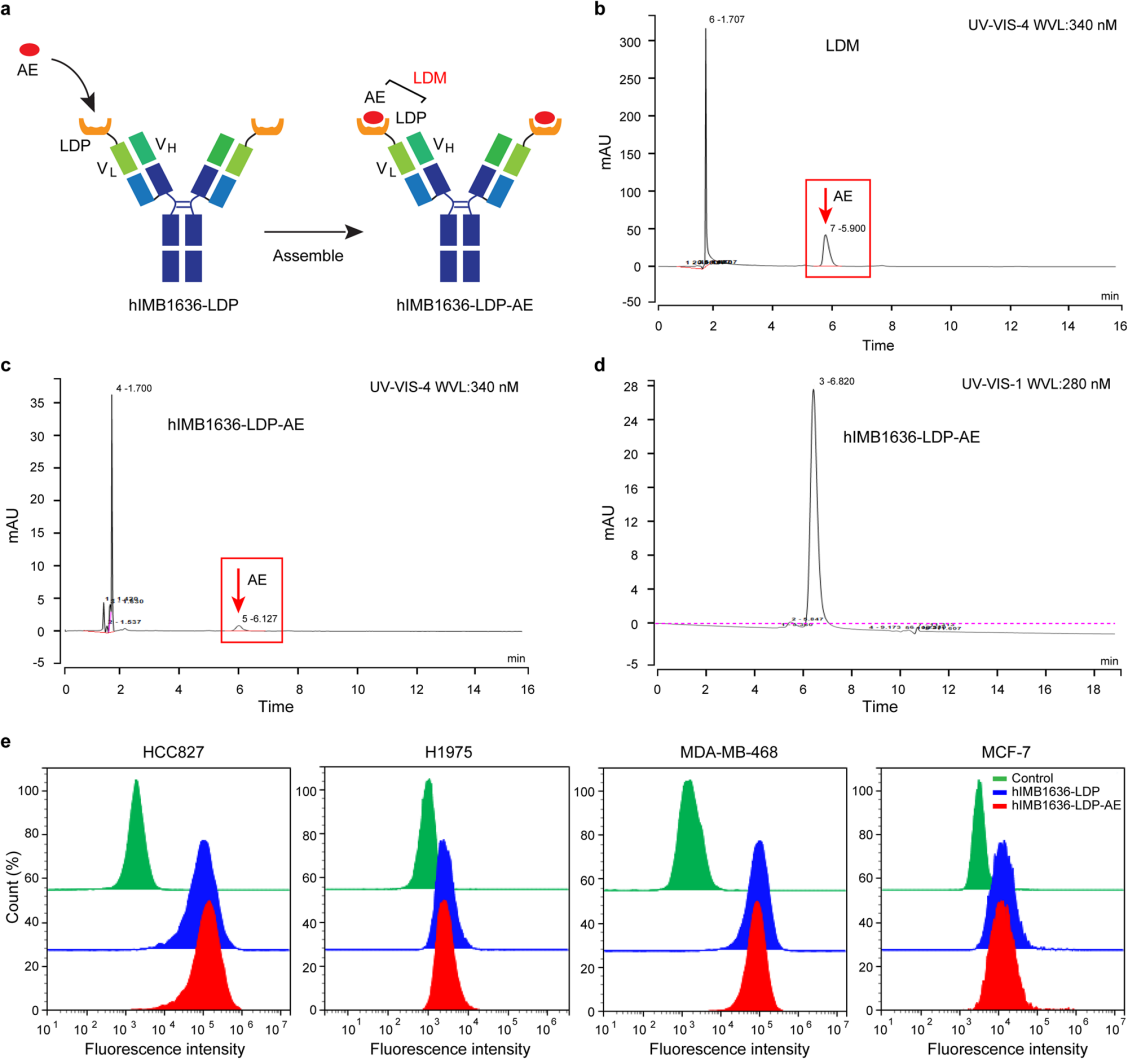
Assembly of hIMB1636-LDP-AE. **a** The schematic diagram of hIMB1636-LDP-AE. **b** Detection of chromophore AE isolated from LDM using a Delta-Pak C4-300A column at 340 nm. **c** HPLC analysis of hIMB1636-LDP-AE using a Delta-Pak C4-300A column at 340 nm. **d** hIMB1636-LDP-AE was analyzed with a BioSep™ SEC-s2000 column at 280 nm. **e** Binding activity of hIMB1636-LDP-AE and hIMB1636-LDP to Trop2^+^ tumor cells by FCM. Data represent mean ± SD, *n* = 3.

We demonstrated the potent affinity and specificity to cell-surface Trop2 antigens by hIMB1636-LDP, which was crucial in performing targeting ability. However, hIMB1636-LDP still required a potent payload to execute cytotoxicity directly. As shown in Fig. 3a, after enediyne chromophore AE was assembled into hIMB1636-LDP, we obtained the fusion protein hIMB1636-LDP-AE containing two LDM molecules. We then separated native LDM molecules into LDP protein and the active enediyne chromophore AE by HPLC and then collected the AE (Fig. 3b). Next, AE and hIMB1636-LDP were assembled to obtain the ADC (hIMB1636-LDP-AE). hIMB1636-LDP-AE was subsequently consecutively examined using a Delta-Pak C4-300A column and molecular sieve SEC-s2000. Our results showed that there was a characteristic peak for AE at 340 nm in the C4-300A chromatogram (Fig. 3c), but not in the molecular sieve chromatogram (Fig. 3d), indicating that there was no free AE mixed in the hIMB1636-LDP-AE solutions and that AE was successfully assembled into the pocket of the LDP protein in hIMB1636-LDP molecules. Additionally, the assembling process appeared to exert almost no influence on the affinity of the antibody as verified by flow cytometry, as the binding activity of hIMB1636-LDP-AE was nearly that for hIMB1636-LDP in HCC827, H1975, MDA-MB-468, and MCF-7 cells (Fig. 3e).

### In vitro antitumor activity of hIMB1636-LDP-AE

**Fig. 4:**
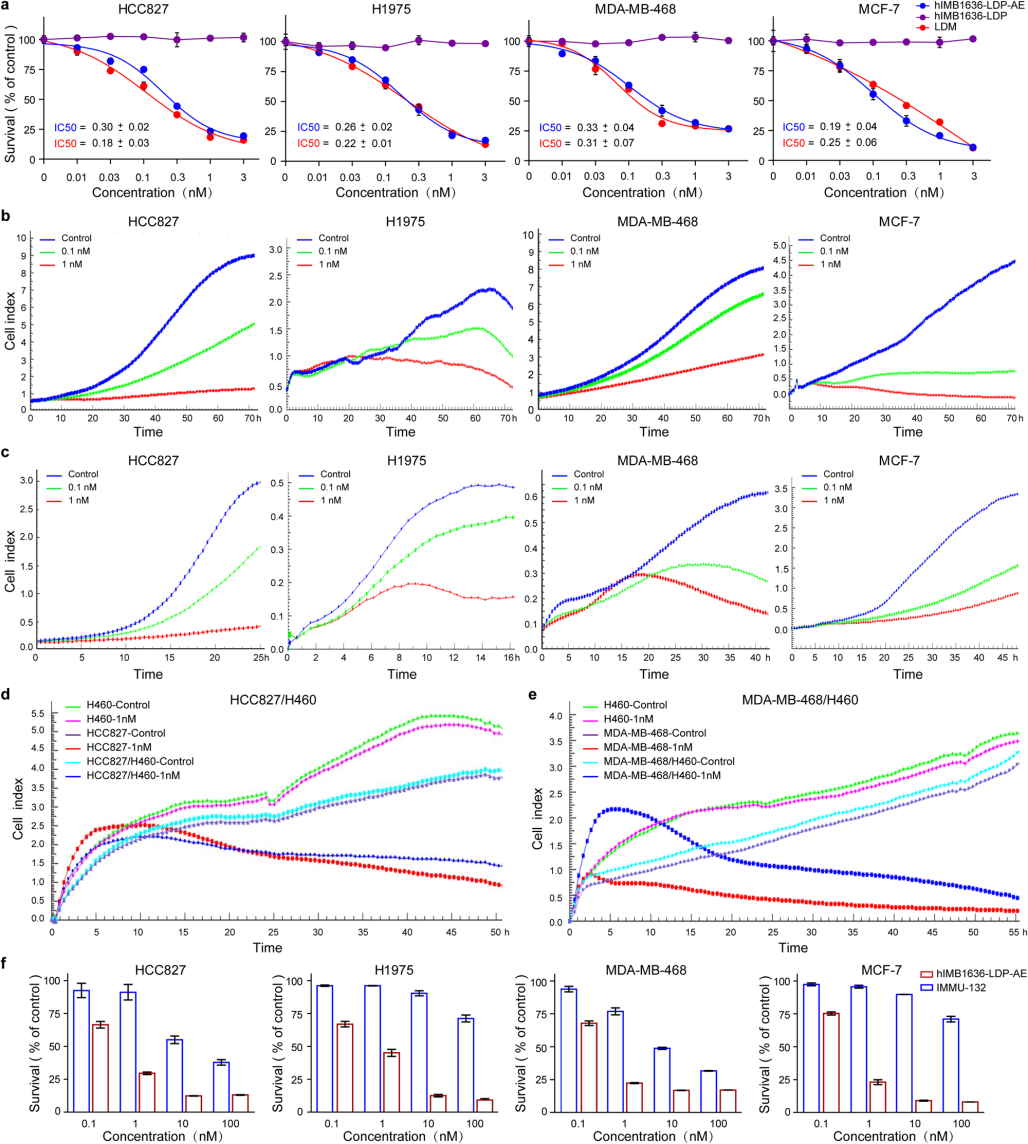
In vitro cytotoxicity of hIMB1636-LDP-AE. **a** Cytotoxicity of hIMB1636-LDP-AE, LDM, and hIMB1636-LDP to different tumor cell types was determined by CCK-8. Data represent mean ± SD, *n* = 3. **b**, **c** Real-time monitoring of proliferation (**b**) and migration (**c**) of HCC827, H1975, MDA-MB-468, and MCF-7 cells after treatment with 0.1 nM or 1 nM hIMB1636-LDP-AE using the xCELLigence system. **d**, **e** Bystander killing effect of hIMB1636-LDP-AE was detected by xCELLigence system. **f** The growth inhibition activity of hIMB1636-LDP-AE and SG against cancer cells.

We evaluated the antitumor effect of hIMB1636-LDP-AE in vitro using the lung cancer cell lines HCC827 and H1975, as well as the breast cancer cell lines MDA-MB-468 and MCF-7, using free LDM as the positive control. Both hIMB1636-LDP-AE and LDM effectively killed the tumor cells, with IC_50_ (the 50% inhibitory concentration) values ranging from 0.18 ± 0.03 nM to 0.33 ± 0.04 nM (Fig. 4a). We also investigated the cytotoxicity of unintegrated hIMB1636-LDP and found that it had no effect on cellular viability (Fig. 4a). We therefore concluded that the cytotoxicity of hIMB1636-LDP-AE was principally due to the action of the AE molecule. Then, the effect of hIMB1636-LDP-AE on the growth and migration of Trop2-positive lung cancer cell lines HCC827 and H1975 were assessed. When we assessed the inverse dose-response correlation between the hIMB1636-LDP-AE concentration and the cell index in Fig. 4b,c, we observed that hIMB1636-LDP-AE significantly inhibited the proliferation and migration of tumor cells in a dose-dependent manner. Compared to the control, 1 nM hIMB1636-LDP-AE showed a cell-proliferation inhibition of 85.97 ± 1.64% and 76.99 ± 0.99% on HCC827 and H1975 cells, respectively, at 72 h (Fig. 4b). The migration inhibition rates of 1 nM hIMB1636-LDP-AE on HCC827 and H1975 cells at 12 h were 86.26 ± 2.25% and 65.04 ± 4.44%, respectively (Fig. 4c), while hIMB1636-LDP-AE also significantly inhibited the proliferation and migration of breast cancer cell lines MDA-MB-468 and MCF-7 in a concentration-dependent manner. The proliferation inhibition rates for 1 nM hIMB1636-LDP-AE were 63.10 ± 2.02% and 87.01 ± 2.47% (Fig. 4b), and the migration inhibition rates were 75.92 ± 1.94% and 73.25 ± 0.92% (Fig. 4c) in MDA-MB-468 and MCF-7 cells, respectively.

We then evaluated the ability of hIMB1636-LDP-AE to induce bystander effect by co-culture of Trop2-negative H460 cells and Trop2-positive cells (HCC827 or MDA-MB-468) using RTCA system. As shown in Figs. 4d, e, 1 nM hIMB1636-LDP-AE treatment almost had no influence on the growth of H460 cells cultured alone, while when co-cultured with HCC827 cells or MDA-MB-468 cells, the growth of H460 cells was delayed significantly. These results suggested that our ADC could kill Trop2-positive cells selectively by receptor-mediated endocytosis and then kill neighboring negative ones by by-stander effects. In addition, we also compared the antitumor effects of hIMB1636-LDP-AE and SG in vitro, and found that the former showed more potent cell-killing ability than the latter (Fig. 4f).

### hIMB1636-LDP-AE inhibits tumorsphere formation

**Fig. 5:**
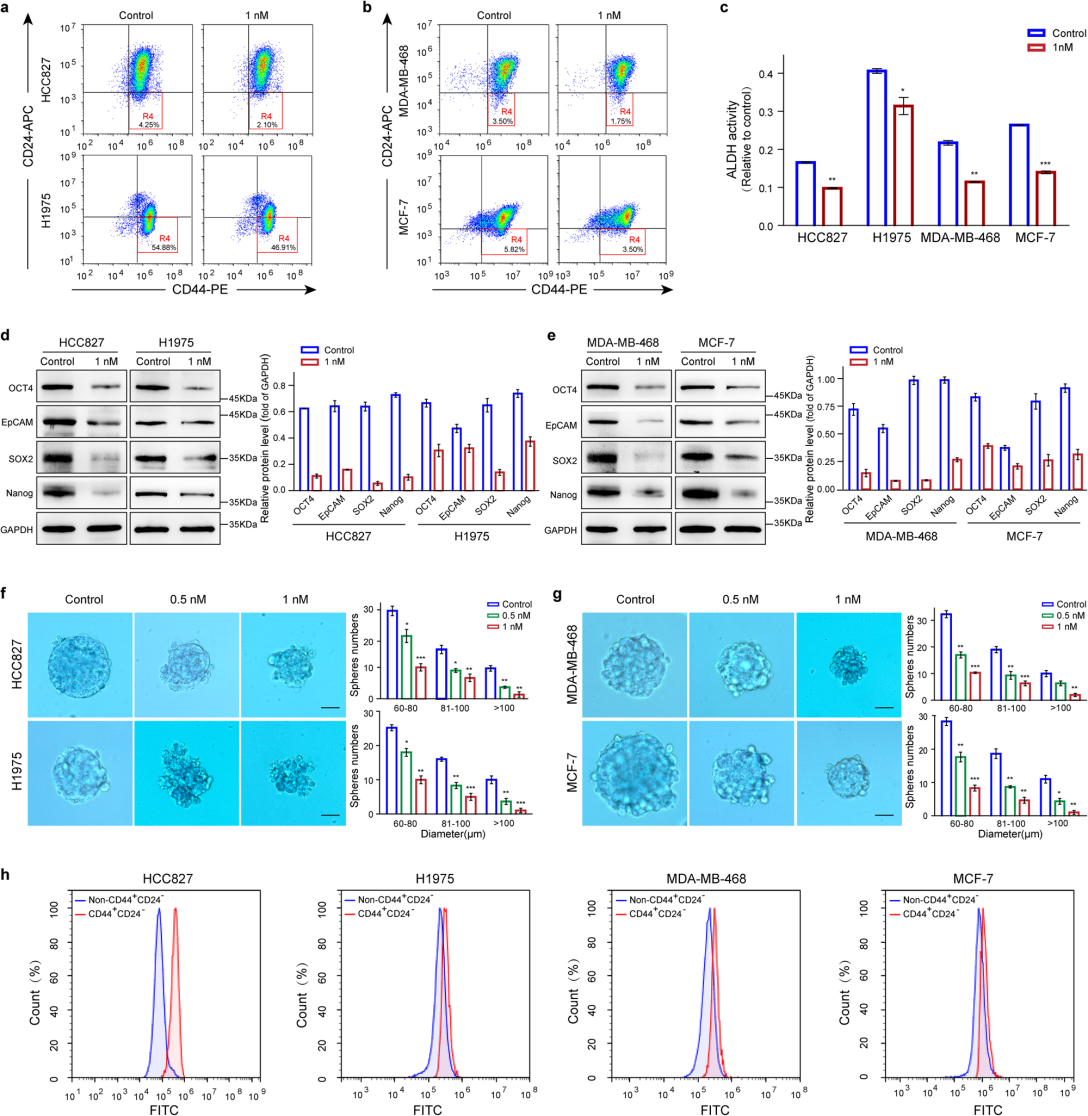
Inhibitory effects of hIMB1636-LDP-AE on CSCs in Trop2^+^ tumor cells. **a** Proportions of subpopulation of CD44^+^/CD24^−^/low CSCs in HCC827 and H1975 cells after treatment with 1 nM hIMB1636-LDP-AE for 24 h. **b** Proportions of subpopulations of CD44^+^/CD24^−^/low CSCs in MDA-MB-468 and MCF-7 cells after treatment with 1 nM hIMB1636-LDP-AE for 24 h. **c** The effects of hIMB1636-LDP-AE on ALDH activity in cancer cells. **d**, **e** Expression of the indicated stem cell marker genes in Trop2^+^ tumor cells treated with 1 nM hIMB1636-LDP-AE by western-blot. Data represent mean ± SD, *n* = 3. Left: Representative images and quantified results of HCC827 and H1975 cells; Right: Representative images and quantified results of MDA-MB-468 and MCF-7 cells. **f**, **g** Tumorspheres formed by HCC827, H1975, MDA-MB-468 and MCF-7cells in 3D culture following hIMB1636-LDP-AE treatment for 10 days. Representative images and the enumeration of tumorspheres are shown. Data represent mean ± SD, *n* = 3. **h** Trop2 expression levels of CD44^high^CD24^low^ cells and non-CD44^high^CD24^low^ cells in different tumor cells. *P < 0.05; **P < 0.01; ***P < 0.001 vs. control. Data represent mean ± SD, *n* = 3.

Cancer stem cells (CSCs) are considered to be the root cause of tumor relapse and resistance. In general, CSCs are identified by CD44^high^CD24^low^ cell surface marker expression^21^. After hIMB1636-LDP-AE treatment, we observed that the proportions of CD44^+^/CD24^−^/low CSCs were reduced not only in HCC827 and H1975 cells (Fig. 5a), but also in MDA-MB-468 and MCF-7 cells (Fig. 5b). Another hallmark of CSCs is their enhanced aldehyde dehydrogenase (ALDH) activity^22^, so we also evaluated the effect of hIMB1636-LDP-AE treatment on ALDH activity in tumor cells. As shown in Fig. 5c, hIMB1636-LDP-AE reduced ALDH activity when compared to the control group, which further indicated the killing effect of hIMB1636-LDP-AE on CSC cells. Moreover, 1 nM hIMB1636-LDP-AE downregulated the protein levels of stem cell markers that included OCT4, SOX2, EpCAM, and Nanog in all four Trop2^+^ tumor cell lines (Fig. 5d, e). As one characteristic of CSCs is their ability to form tumorspheres^23^, we subsequently evaluated the effect of hIMB1636-LDP-AE on tumorsphere formation of Trop2^+^ tumor cells. As shown in Fig. 5f, g, tumor spheroids with well-defined smooth edges were observed in control cells with PBS; while in cells exposed to hIMB1636-LDP-AE, the spheroids had jagged, rough edges relative to the controls and were not as round. Compared with the controls, the numbers of tumorspheres larger than 100 μm were reduced by 82.76.0 ± 3.2% (*P* < 0.01) and 87.88 ± 2.6% (*P* < 0.01) in HCC827 and H1975 cells treated with 1 nM hIMB1636-LDP-AE, respectively (Fig. 5f). Similarly, in MDA-MB-468 and MCF-7 cells, the number of tumorspheres also decreased by 90.00 ± 0.2% (*P* < 0.01) and 80.00 ± 0.7% (*P* < 0.01), respectively (Fig. 5g). Furthermore, we observed that cancer cells after hIMB1636-LDP-AE treatment lost the potential to form secondary spheres (Supplementary Fig. S1). All of the above results showed that the hIMB1636-LDP-AE prevented the development of the CSCs into tumorspheres and actually exerted a killing effect on them. More importantly, we found that the expression level of Trop2 in CD44^high^CD24^low^ cells was higher than that of non-CD44^high^CD24^low^ cells, which might just explain the potential killing effect of hIMB1636-LDP-AE on cancer stem cells (Fig. 5h).

### hIMB1636-LDP-AE induces cell apoptosis and cycle arrest

**Fig. 6:**
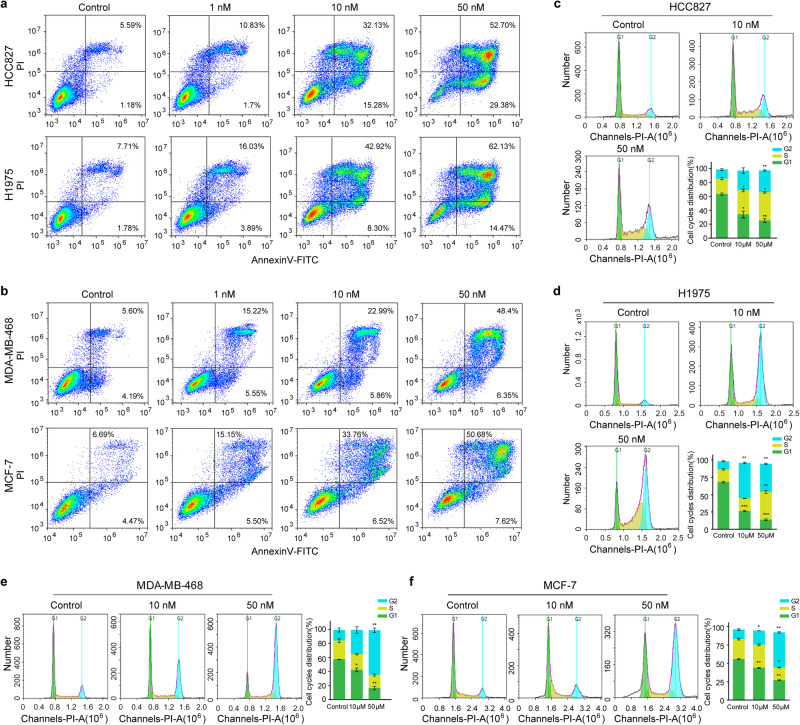
hIMB1636-LDP-AE induces apoptosis and cell-cycle arrest. **a** Apoptosis analysis of HCC827 and H1975 cells after treatment with the indicated concentration of hIMB1636-LDP-AE for 24 h by FCM. **b** Apoptosis analysis of MDA-MB-468 and MCF-7 cells after treatment with indicated concentration hIMB1636-LDP-AE for 24 h by FCM. **c**, **d** Cell-cycle phase distribution and quantitative analysis in HCC827 (**c**) and H1975 (**d**) cells after treatment with the indicated concentration of hIMB1636-LDP-AE for 24 h. Data represent mean ± SD, *n* = 3. **e**, **f** Cell-cycle phase distribution and quantitative analysis in MDA-MB-468 (**e**) and MCF-7 (**f**) cells after treatment with the indicated concentration of hIMB1636-LDP-AE for 24 h. *P < 0.05; **P < 0.01; ***P < 0.001 *vs*. control. Data represent mean ± SD, *n* = 3.

Apoptosis induction and cell cycle arrest are two important causes of cell death. To explore the molecular mechanism(s) underlying hIMB1636-LDP-AE action on the inhibition of cell growth and proliferation, we executed flow cytometry to detect the molecule’s effect on apoptosis and cell cycle kinetics. Our results revealed that hIMB1636-LDP-AE induced both early and late apoptotic changes in a dose-dependent manner compared to the controls in these tumor cells (Fig. 6a, b), with 50 nM hIMB1636-LDP-AE increasing the percentages of late apoptotic cells to 52.70 ± 1.28% and 62.13 ± 1.03% in HCC827 and H1975 cells, respectively (Fig. 6a). hIMB1636-LDP-AE also increased apoptosis in both MDA-MB-468 and MCF-7 cells in a concentration-dependent manner (Fig. 6b). In contrast, we also evaluated the cell cycle distribution in Trop2^+^ tumor cell lines treated with different concentrations of hIMB1636-LDP-AE and observed a significant decrease in G1 DNA content and an increase in G2 DNA content in a concentration-dependent manner, not only in HCC827 and H1975 cells (Fig. 6c, d) but also in MDA-MB-468 and MCF-7 cells (Fig. 6e, f). These results indicated that hIMB1636-LDP-AE exerted its antitumor effect by inducing apoptosis and cell-cycle arrest at the G2 phase.

### hIMB1636-LDP-AE inhibits tumor growth in vivo

**Fig. 7:**
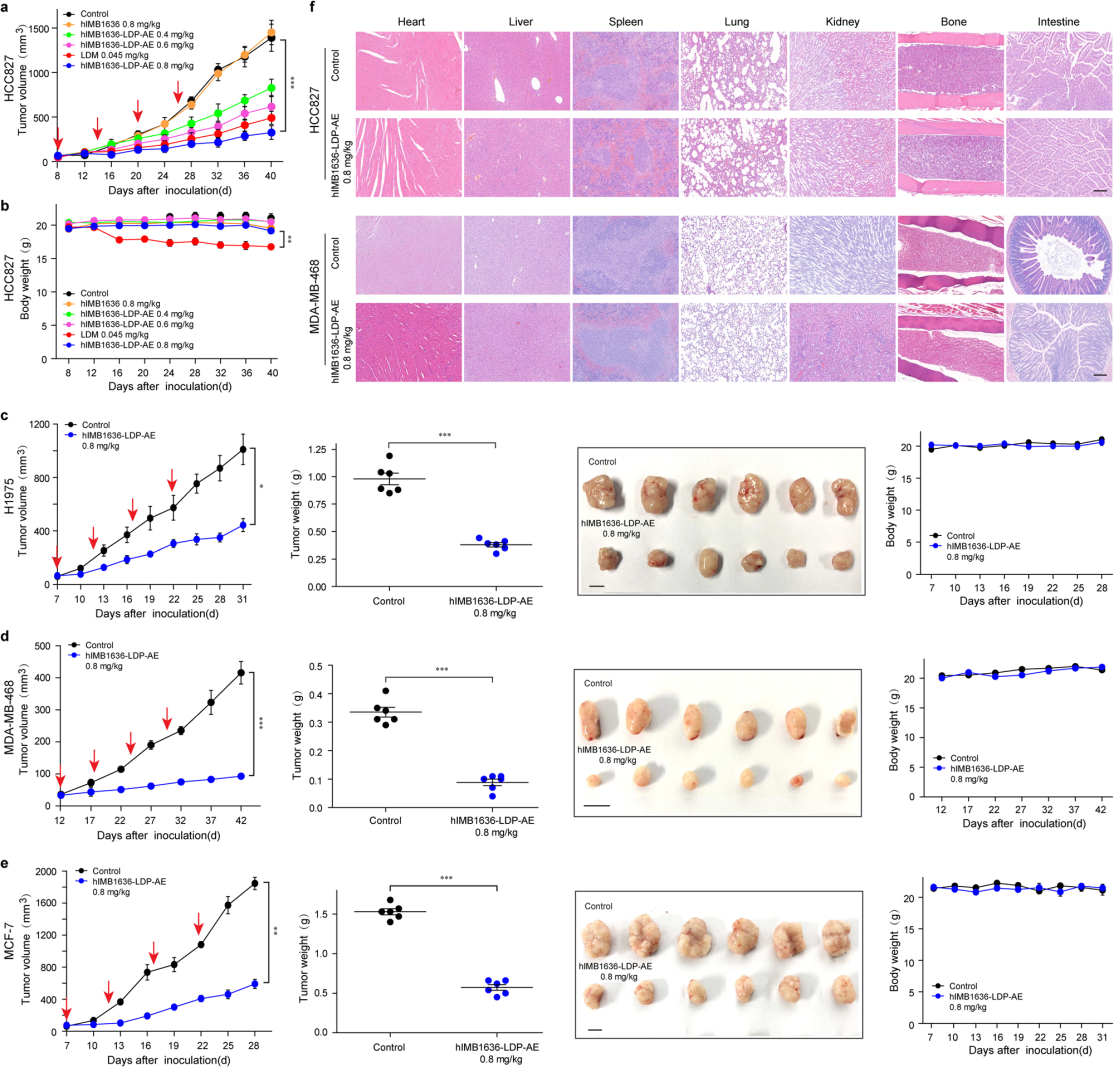
Therapeutic efficacy of hIMB1636-LDP-AE against Trop2+ tumor cells in vivo. **a** Growth curves of HCC827 cell-derived xenograft tumors under different treatments. **b** Body weight curves of mice as described in a. **c** Tumor growth curve, tumor weight, and tumor images of H1975 cell-derived xenograft tumors, and body weight curves of mice. **d** Tumor growth curve, tumor weight, tumor images of MDA-MB-468 cell-derived xenograft tumors, and body weight curves of mice. **e** Tumor growth curve, tumor weight and tumor images of MCF-7 cell-derived xenograft tumors, and body weight curves of mice. **f** H&E staining of various organs and tumors of HCC827 and MDA-MB-468 xenograft-bearing mice treated with hIMB1636-LDP-AE at a dose of 0.8 mg/kg. Magnification, 100×. *P < 0.05; **P < 0.01; ***P < 0.001 vs. control. Data represent mean ± SD, *n* = 6.

Using a subcutaneous xenograft model, we evaluated the antitumor activity of hIMB1636-LDP-AE in vivo. A HCC827 xenograft model was first established, and we initiated treatment when tumor volumes reached approximately 80 mm^3^. We noted that hIMB1636-LDP-AE inhibited the growth of HCC827 xenografts in a concentration-dependent manner, and that at 0.8 mg/kg, hIMB1636-LDP-AE generated a nearly 76.62 ± 10.99% tumor inhibition rate, which was higher than the 64.75 ± 10.55% inhibition rate for free LDM at the maximally tolerated dose; while free antibody at 0.8 mg/kg showed no antitumor effects with tumor volumes similar with that of the control group (Fig. 7a). We additionally did not observe death or significant adverse effects, except for significant weight loss in the LDM-treated group (*P* < 0.01 *vs*. hIMB1636-LDP-AE at 0.8 mg/kg, Fig. 7b). We then explored the therapeutic potential of 0.8 mg/kg hIMB1636-LDP-AE on other Trop2-positive xenograft models. As expected, compared with the control group, hIMB1636-LDP-AE treatment significantly reduced tumor growth, tumor weight, and tumor size, with a tumor inhibition rate of 56.15 ± 10.85%, 77.59 ± 1.78%, and 67.94 ± 6.91% in H1975, MDA-MB-468, and MCF-7 xenograft models, respectively (Fig. 7c–e); however, no visible body weight change was noted for any treatment group (Fig. 7c–e). More importantly, we observed no significant toxico-pathological changes in the heart, liver, spleen, lung, kidney, bone marrow, or small intestine of mice treated with 0.8 mg/kg hIMB1636-LDP-AE (Fig. 7f) in the HCC827 and MDA-MB-468 xenograft models, suggesting that our administered doses exerted no toxic side-effects.

**Fig. 8:**
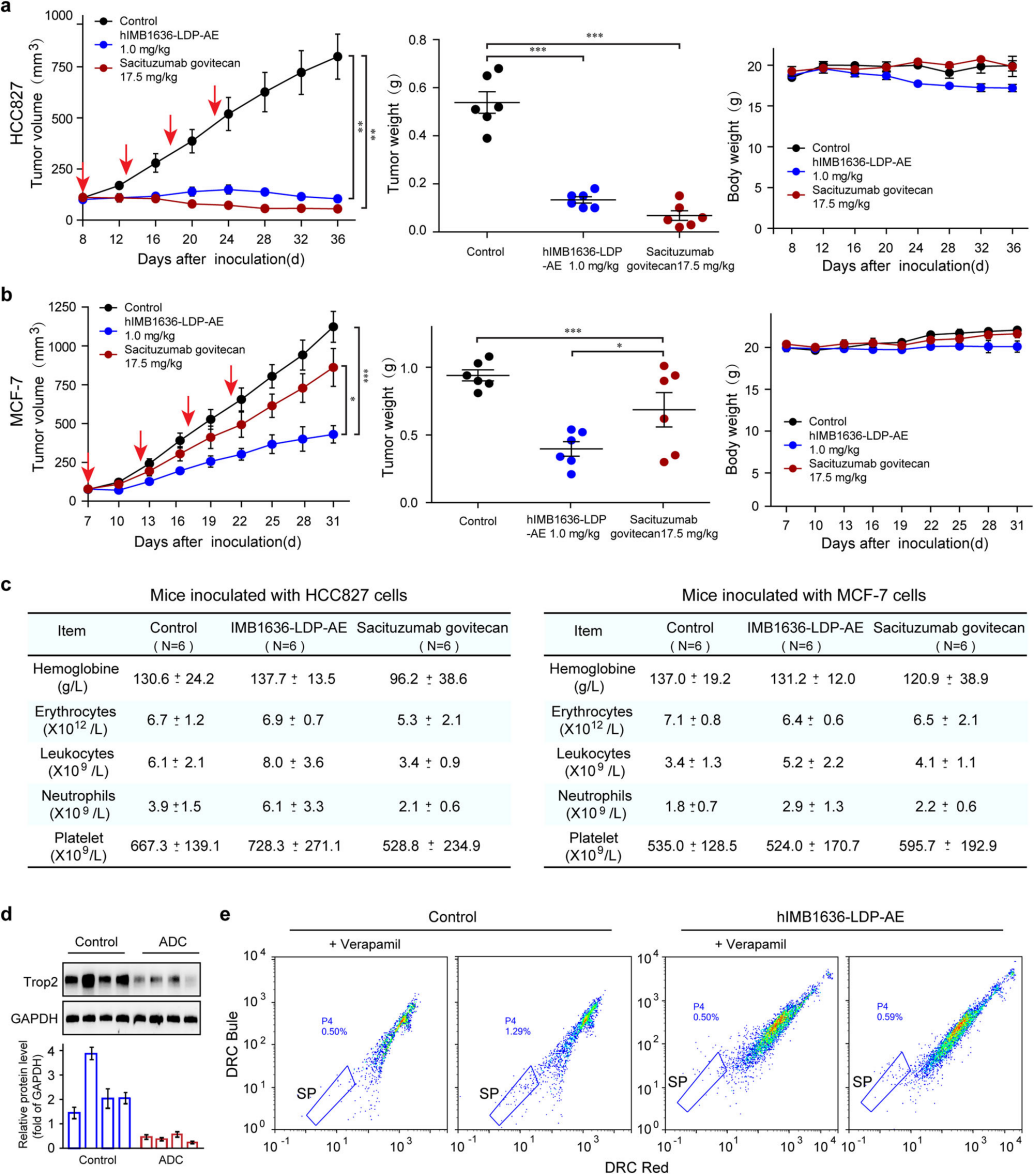
Therapeutic efficacy of hIMB1636-LDP-AE and SG against Trop2^+^ tumor cells in vivo. **a** Tumor growth curve, tumor weight of HCC827 cell-derived xenograft tumors, and body weight curves of mice. **b** Tumor growth curve, tumor weight of MCF-7 cell-derived xenograft tumors, and body weight curves of mice. **c** Haematological parameters of mice under different treatments. **d** Detection of Trop2 protein expression in HCC827 cell-derived tumors by western-blot. Data represent mean ± SD, *n* = 3. **e** SP cell analysis of tumors from HCC827 xenograft-bearing mice under different treatments. *P < 0.05; **P < 0.01; ***P < 0.001 *vs*. control. Data represent mean ± SD, *n* = 6.

Subsequently, we compared the anti-tumor effects of hIMB1636-LDP-AE and SG in vivo, and the results showed that both induced significant and apparently similar anti-tumor effects in HCC827 (high Trop2 expression) xenograft model (Fig. 8a). However, in MCF-7 (moderate Trop2 expression) xenograft model, hIMB1636-LDP-AE displayed significantly more potent tumor inhibition effects than SG (hIMB1636-LDP-AE *vs*. SG *P* = 0.027, Fig. 8b), indicating that hIMB1636-LDP-AE might have better therapeutic effects against tumors with moderate Trop2 expression. In addition, hIMB1636-LDP-AE therapy displayed no obvious effects on hematological parameters such as hemoglobine, leukocyte-, platelet- and neutrophil counts, which indicated that our ADC had no significant myelotoxicity (Fig. 8c). However, mice treated with SG experienced apparent reduction of red blood cells, white blood cells and platelets in HCC827 xenograft model (Fig. 8c), which was consistent with previous reports that myelosuppression was one of the primary treatment-related adverse events in SG^24^. Importantly, the expression level of Trop2 in tumors treated with hIMB1636-LDP-AE was significantly lower than that in control group (Fig. 8d), which further confirmed the specificity of hIMB1636-LDP-AE for Trop2 antigen in vivo. The side population (SP) assay has been utilized as a method for isolation and characterization of normal and cancer stem cells^25^. Surprisingly, compared with control group, the proportion of SP cells in tumor samples from mice treated with hIMB1636-LDP-AE was significantly reduced (0.59 vs.1.29, Fig. 8e), which further indicated that it owned the ability to kill cancer stem cells in vivo.

## Discussion

Lung cancer and breast cancer are the most common malignant diseases worldwide. The 5-year survival rate (SR) of lung cancer patients is only 4–17%^26^. Breast cancer tends to spread to distant sites, including the lung, liver, bone, and brain. Of these, 60–70% of metastatic breast cancer patients who eventually died were diagnosed with lung metastasis. The 5-year SR of breast cancer patients with distant metastasis is also less than 20%^27,28^. Trop2 is a membrane protein that is highly expressed in many tumors, including breast, lung, gastric, colorectal, pancreatic, prostatic, and ovarian carcinomas, making it a potential and attractive target for ADCs^29,30^. Based on the specificity of monoclonal antibodies (mAbs) to recognize the cell-surface antigens of cancer, ADCs can deliver toxic payload directly to the tumor site, thereby reducing the toxic effect in normal tissues^31^. In 2020, the first ADC-targeting Trop2, IMMU-132, which used the active metabolite SN-38 of irinotecan as the payload, was approved in the USA^32^, prompting more research on the Trop2 signaling pathway and the development of anti-Trop2 ADCs. Irinotecan is a common chemotherapeutic agent which has been widely used for many years in cancer treatment^33^. Irinotecan acts through its active metabolite SN-38 as an inhibitor of topoisomerase I, resulting in DNA breaks and the suppression of DNA synthesis, and ultimate cancer cell death. However, intrinsic and acquired resistance to irinotecan constitutes a major clinical problem^34^. Notably, IMMU-132, with SN-38 as the toxic payload, will inevitably be affected by this resistance. A study has found that irinotecan-resistant tumor cell lines have a certain resistance to IMMU-132, which weakens its tumor-inhibitory effect^35^. This highlights the need to study new ADCs with different mechanisms of action from SN-38 to treat Trop2^+^ tumors.

In this study, we generated a new type of ADC hIMB1636-LDP-AE by combining the cytotoxic molecule LDM with the anti-Trop2 antibody hIMB1636 via a non-cleavable peptide linker. LDM, developed by our institute, is a member of the enediyne antitumor antibiotic family. The potential clinical value of enediyne antibiotics is also confirmed by the approval of Besponsa and Mylotarg, immunoconjugates that use calicheamicin, another member of the enediyne-containing family, as cytotoxic payloads^36,37^. Like other members of the enediyne antitumor antibiotic family such as calicheamicin and neocarzinostatin, LDM exerts extreme cytotoxic potencies by abstracting hydrogen atoms from carbons of the sugar backbone in the minor groove of complementary DNA, leading to double-strand or single-strand breaks of DNA molecules, in the presence of oxygen^38^. The hypoxic microenvironment in solid tumors limits the effects of anti-cancer therapies like ionizing radiation and conventional radiomimetics such as enediynes which need oxygen to cause lethal DNA breaks^39^. However, LDM can induce inter-strand cross-links of DNA instead of DNA breaks in the hypoxic tumor microenvironment; this unique mechanism of action makes it nearly three-fold more cytotoxic to hypoxic than to normoxic cells^40^. Thus, LDM can preferentially target hypoxic cells and defeat radioresistance related to hypoxia in cancer cells. Furthermore, LDM is also highly potent to multidrug-resistant cancer cells^41^. In addition, the phase I clinical trials of LDM have been completed, and no immunogenicity-related issues have been found. Therefore, LDM is an ideal payload for the preparation of ADCs. The present data indicate that hIMB1636-LDP-AE exerts cytotoxic effects by inducing DNA damage, cell cycle arrest, and apoptosis, with a mechanism similar to that of LDM. Taken together, we speculated that hIMB1636-LDP-AE might have the potential to overcome resistance induced by chemotherapy, radiotherapy, and other types of anti-Trop2 ADCs.

In the present study, hIMB1636-LDP-AE was developed using a unique two-step method according to the attractive property in which LDM could be dissociated and reconstituted in vitro^42^. First, the fusion protein hIMB1636-LDP, an anti-Trop2 antibody hIMB1636 fused with the apoprotein LDP of LDM, was generated through genetic engineering. Second, hIMB1636-LDP-AE was prepared by integrating the enediyne chromophore AE into the fusion protein hIMB1636-LDP via molecular reconstitution. Compared with complex chemical processes such as the modifications of antibodies and payloads as well as the synthesis of linkers and linker-payloads in the preparation of traditional ADCs, the present process is simple and convenient. Of importance, most ADCs were synthesized by traditional non-specific chemical conjugation methods using lysines or cysteines on the antibody surface linked with payload via amino or sulfhydryl-specific linkers, resulting in irreproducible processes and heterogeneous products that vary in both coupling sites and drug-to-antibody ratios (DARs). Heterogeneous ADCs contain a variety of components with suboptimal DARs, which are known to have unanticipated pharmacological properties^43,44^. In contrast, our ADC showed a well-defined product with a DAR of 2:1, because the LDP molecule was site-specifically linked to the N-terminal of the light chain in the antibody via a peptide linker. These properties suggest that our ADC products may be more homogeneous, thus facilitating quality control during their manufacturing process.

To verify the affinity and targeting of hIMB1636-LDP-AE, both in vitro and in vivo studies were conducted. The results of SPR analysis have shown that the K_D_ value of hIMB1636-LDP was 4.6 nM, slightly increased in comparison with that of naked antibody (0.6 nM) (Fig. 1d, e). Notably, fusion protein hIMB1636-LDP showed almost the same binding activities to different cancer cells with naked antibody hIMB1636 (Fig. 2a), indicating the connection of apoprotein LDP with the parent anti-Trop2 antibody exerted a minimal adverse effect on the binding affinities of hIMB1636 antibody. Both flow cytometry and confocal imaging analysis demonstrated that our ADC can be internalized and trafficked to lysosomes by Trop2-positive cancer cells. In vivo imaging analysis revealed that both hIMB1636-LDP and naked hIMB1636 antibodies were specifically targeted to the tumor within 6 h and continued to accumulate at the tumor site for more than 10 days. Together, these data indicated that linking LDP with the hIMB1636 antibody cannot affect the activities of the parent antibody. Furthermore, hIMB1636-LDP was assembled with active chromophore AE separated from LDM, and hIMB1636-LDP-AE was successfully obtained. The results of our cytotoxic assay showed that both hIMB1636-LDP-AE and LDM showed potent efficacies with IC_50_ values at the sub-nanomolar level against different kinds of cancer cells, and the hIMB1636-LDP did not affect the survival of tumor cells, suggesting that AE is the main cytotoxic moiety in the hIMB1636-LDP-AE molecule responsible for killing tumor cells.

Additionally, hIMB1636-LDP-AE also significantly inhibited the proliferation and migration of Trop2-positive tumor cells in a dose-dependent manner. More importantly, hIMB1636-LDP-AE owns the ability to induce bystander-killing effect, which indicates that it could kill Trop2-negative tumor cells in tumor microenvironment and overcome heterogenecity of tumors to a certain degree. CSCs, which possess self-renewal and multilineage differentiation capacities, are considered to be an engine of tumor evolution^45,46^. hIMB1636-LDP-AE also strongly inhibited the function of CSCs, as evidenced by the decreases in tumorsphere formation and reduced expression levels of CSC-related markers such as OCT4, SOX2, EpCAM, and Nanog (Fig. 5d-g). Hence, hIMB1636-LDP-AE can not only kill tumor cells but also demonstrate a powerful killing ability against tumor stem cells. Moreover, hIMB1636-LDP-AE inhibited the growth of cancer cells by inducing apoptosis and cycle arrest. In addition, in vivo antitumor study further confirmed that hIMB1636-LDP-AE improved antitumor efficiency compared with naked anti-Trop2 antibody or free lidamycin alone (inhibition rate: 76.62 ± 10.99% vs. 64.75 ± 10.55%). On the other hand, hIMB1636-LDP-AE exhibited a favorable safety profile by histopathological examination, as no visible lesions were observed in various organs of treated mice at therapeutic doses.

Given that SG has been approved by US FDA for the therapy of patients with metastatic triple-negative breast cancer, we also compared the antitumor effects of hIMB1636-LDP-AE and SG. The results showed that both almost completely inhibited the growth of HCC827 tumors (high Trop2 expression), while hIMB1636-LDP-AE showed more potent antitumor effects than SG against MCF-7 xenograft model (moderate expression). Considering that ADCs entered cancer cells by receptor-mediated endocytosis, maybe the limited Trop2 expression in MCF-7 cells restricted numbers of ADC molecules that entered the cells. While lidamycin demonstrated 100 times or more potent cell-killing activity than SN-38 in vitro, which might explain why hIMB1636-LDP-AE displayed more potent antitumor activity in MCF-7 xenograft model^47–50^. These results were consistent with the reports that the use of ADCs bearing more highly potent payloads would increase the probability of delivering a therapeutic dose to tumor cells with low antigen expression^51^. Furthermore, although myelosuppression was one of the primary treatment-related adverse events in SG^24^, no obvious myelotoxicity was observed in hIMB1636-LDP-AE-treated mice. Notably, the fusion of LDM to hIMB1636 mAb improved the tolerability and tumor-therapeutic efficacy of LDM in nude mice, and suggesting that hIMB1636-LDP-AE may constitute a promising therapeutic candidate against Trop2-expressing tumors.

In this study, we designed and developed a new ADC, hIMB1636-LDP-AE, through genetic engineering and molecular-recombination techniques. The ADC displayed specific affinity, targeting, and potent antitumor as well as anticancer stem cell activities in breast cancer and lung cancer both in vivo and in vitro. Moreover, compared to SG, it exhibited more potent antitumor effects and significantly lower myelotoxicity in tumors with moderate Trop2 expression. The preclinical results presented here, together with our unique preparation process, suggested that hIMB1636-LDP-AE may constitute a promising candidate for the treatment of breast and lung cancers.

## Methods

### Reagents

Recombinant human Trop2 protein (10428-H08H) was purchased from Sino Biological (Guangzhou, China). DAPI (ZLI-9557) was obtained from ZSGB-Bio (Beijing, China). TMB substrate (PA107) was obtained from Tian Gen (Beijing, China). A CCK-8 assay kit (CK04) was purchased from Dojindo (Tokyo, Japan). StemXVivo Tumor Sphere Media (CCM012) was obtained from R&D Systems (Minnesota, USA). The BCA protein assay kit (PC0020) and RIPA lysis buffer (R0010) were obtained from Solarbio (Beijing, China). Cell cycle kits (CCS012) and Annexin V-FITC/PI apoptosis kits (AP101) were purchased from Multi Sciences (Zhejiang, China). Verapamil (S4202) was obtained from Selleck.cn. Vybrant^TM^ DyeCycle^TM^ Violet Ready Flow^TM^ (DCV, R37172) were purchased from Thermo Fisher Scientific (Waltham, USA). Cell culture medium was obtained from Basal Media (Shanghai, China), and Fetal Bovine Serum (FBS) was purchased from VivaCell Biosciences (Shanghai, China).

### Cells and cell culture

A lung cancer cell line (HCC827) and breast cancer cell lines (MDA-MB-468, MCF-7) were obtained from the Cell Center of Peking Union Medical College (Beijing, China). Lung cancer cell lines (H1975, A549, H460) and breast cancer cell line MDA-MB-231 were preserved in our laboratory. MDA-MB-468, MDA-MB-231, and MCF-7 cells were cultured in a DMEM medium supplemented with 10% FBS. Other cells were cultured in RPMI-1640 medium supplemented with 10% FBS. All cell cultures were incubated at 37 °C in a 5% CO_2_ atmosphere.

### Construction and expression of hIMB1636-LDP fusion proteins

In previous studies, we obtained the sequence of variable regions (VH and VL domains) of the hIMB1636 antibody by hybridoma sequencing (unpublished data). The expression vector pIZDHL, which carries the gene sequence encoding the human IgG1 constant region, was preserved by our laboratory. The construction and expression of recombinant protein were performed as previously described. Briefly, the C-terminal LDP protein of LDM was designed to link with the N-terminal of VL of hIMB1636 via a non-cleavable peptide linker (SGGPEGGS) to obtain an expression vector. DNA fragments encoding the VH and LDP-SGGPEGGS-VL protein sequences were synthesized by GenScript company (Nanjing, China) and then ligated into the vector pIZDHL by standard subcloning methods. Furthermore, the expression vector, named pIZDHL-hIMB1636-LDP, was obtained as previously described^49^. As the control, VH and VL sequences were also ligated into the pIZDHL vector for the expression of parental hIMB1636 antibody, and the pIZDHL-hIMB1636 expression vector was obtained. Then, the pIZDHL-hIMB1636-LDP and pIZDHL-hIMB1636 expression vectors were linearized and transfected into CHO/dhFr^-^ cells by Lipofectin transfection (Invitrogen, CA, USA) to generate cell lines with hIMB1636-LDP and hIMB1636 antibody expression, respectively. Next, the cells were cultured and sub-cloned with the selective medium as previously described^49^. Finally, the clones producing the highest levels of fusion proteins and antibodies were selected for further study.

### Purification and purity analysis of the recombinant proteins

The selected cell clones were expanded cultures for antibody preparation. First, cells were cultured for 24 h, and then the culture medium was changed to serum-free CHO medium (Bioengine, Shanghai, China) with GlutaMAX^TM^ supplement (Gibco, New York, USA). After 10 days, the cell culture medium was collected and the recombinant protein hIMB1636-LDP and hIMB1636 antibody were purified by HitrapTM protein G columns (GE Healthcare, Chicago, USA) according to the manufacturer’s instructions. In brief, the cell culture medium was passed through the protein G column, the recombinant protein was bound to the column, and then eluted with elution buffer at pH 2.5. The collected protein was desalinated and quantified by the BCA protein assay kit (Thermo Fisher Scientific, Waltham, USA). The purified proteins were investigated by non-reducing and reducing SDS-PAGE gels. An uncropped scan of the gel is shown in Supplementary Fig. S2.

### Enzyme-Linked Immunosorbent Assay (ELISA)

The binding ability of anti-Trop2-LDP and anti-Trop2 antibody with antigen were determined by ELISA. Trop2 protein (0.2 μg/well) was added to a 96-well plate and incubated overnight at 4 °C. A blocking buffer (5% BSA in PBS) was added to the 96-well plate to block nonspecific binding after washing with PBST. After blocking for 2 h, hIMB1636-LDP or hIMB1636 antibody of different concentrations was added to the respective wells and incubated at 37 °C for 2 h. The wells were then washed with PBST and incubated with HRP-labeled goat anti-human IgG dilute at 1:2000 for 1 h. Finally, TMB was added for color reaction, and then 2 M sulfuric acid was added to stop the reaction. The optical density at 450 nm was measured using a microplate reader (Molecular Devices, Austria).

### Surface plasmon resonance analysis

A Biacore T200 SPR instrument (GE Healthcare, Chicago, USA) was used to examine the SPR reaction of Trop2 antigen with anti-Trop2-LDP or anti-Trop2 antibody (as control). The CM5 sensor chip was immobilized with Trop2 antigen. Various concentrations of hIMB1636-LDP or hIMB1636 antibody in HEPES buffer were injected at a flow rate of 30 μL/min. After each detection cycle, the sensor surface was regenerated with 3 M MgCl_2_ (flow rate, 30 μL/min; contact time, 60 s), allowing resonance signals to return to baseline values. We ultimately applied Biacore T200 evaluation software for data processing and analysis.

### Protein extraction and western-blot analysis

To assess Trop2 protein expression in different cell lines, we cultured and collected a variety of tumor cells that included HCC827, H460, H1975, A549, MCF-7, MDA-MB-468, and MDA-MB-231 cells. These cells were lysed with RIPA lysis buffer, and the cellular protein was extracted and its concentration determined using a BCA protein assay kit. The protein samples were then separated on SDS-PAGE, followed by transferring them onto polyvinylidene difluoride membranes. After blocking, the membranes were probed with specific primary and secondary antibodies. The protein bands were detected with an ECL detection system (Tanon, Shanghai, China). Antibodies against Trop2 (Sino Biological, 10428-MM01, 1:1000), GAPDH (ZSGB-Bio, TA-08, 1:1000), and the secondary antibodies conjugated to HRP (ZSGB-Bio, ZB-2301 or ZB-2305,1:2000) were used. Uncropped scans of western blots were provided in Supplementary Fig. S3.

### Flow-cytometric analysis

The binding of hIMB1636-LDP and hIMB1636 antibody to native Trop2 antigen on the surface of tumor cells was detected by flow cytometry. Cells were collected (5 × 10^6^/tube) and 5 μg/mL hIMB1636-LDP or 5 μg/mL hIMB1636 was added to the tubes and incubated at 4 °C for 2 h. After washing with PBS three times, a FITC-labeled secondary antibody was added and incubated at 4 °C for 1 h in the dark. The labeled cells were washed, and cell-associated fluorescence signals were determined by flow cytometer (FCM) (ACEA Biosciences Inc., California, USA). The FACS gating strategies are given in Supplementary Fig. S4.

### Analysis of hIMB1636-LDP internalization

To detect internalization of hIMB1636-LDP by flow cytometry, the cells were collected (5 × 10^6^/tube) and incubated for 0.5 h with 5 μg/mL hIMB1636-LDP protein at 4 °C. The cells were then partitioned into two groups. One group served as a control for total cell surface binding and continued to be incubated at 4 °C for 2 h. For the other group, internalization was assessed upon incubation at 37 °C for 2 h. Next, all cells were washed three times with PBS. After washing, the cells were incubated with FITC-labeled secondary antibody at 4 °C for 1 h under dark conditions. The labeled cells were washed, and cell-associated fluorescence signals were determined by FCM. The FACS gating strategies are shown in Supplementary Fig. 3.

For confocal internalization analysis, the cells were seeded on coverslips in 24-well plates and cultured for 48 h, and then incubated with hIMB1636-LDP antibody (5 μg/well) at 4 °C for 0.5 h. Some of these cells were subsequently incubated at 37 °C for 2 h, while other cells were incubated at 4 °C as a control. Next, all cells were fixed in 4% paraformaldehyde, permeabilized with 0.5% Triton X-100, and blocked with 5% BSA for 0.5 h at room temperature. The cells were stained with the anti-LAMP1 antibody (Cell Signaling Technology, 9091 S, 1:1000) and incubated overnight at 4 °C. The corresponding fluorescein-labeled secondary antibodies were then added and incubated at 37 °C for 2 h under dark conditions (i.e., the anti-rabbit Alexa Fluor 555 antibody (Invitrogen, A-31572,1:100) was used to label the LAMP-1 on lysosomes and anti-human FITC antibody (ZSGB-Bio, ZF-0308,1:100) to label hIMB1636-LDP). The nuclei were counterstained with DAPI, and the images were captured with a confocal fluorescence microscope (Olympus Microsystems, California, USA).

### In vivo imaging of fluorescein-labeled hIMB1636-LDP

The in vivo tumor-targeting ability of hIMB1636-LDP was investigated using HCC827 and MDA-MB-468 xenograft tumor models in BALB/c nude mice. When the solid tumors attained a volume of approximately 200 mm^3^, anti-Trop2-LDP was labeled with DyLight 680 dyes according to the manufacturer’s instructions and injected into mice through their tail veins at a dose of 20 mg/kg. The mice were then placed in the imaging chamber of a Xenogen IVIS-200 system (Xenogen Inc., California, USA) for in vivo distributional observations at a series of time points after anesthetization with isoflurane. Finally, the mice were sacrificed using CO_2_ inhalation, and the solid tumors and other normal tissues (including heart, liver, spleen, lung, and kidney) were removed and photographed. The images were analyzed with Living Image software (Xenogen Inc., California, USA).

### Assembly of hIMB1636-LDP-AE

The chromophore AE of LDM was separated through a Delta Pak C4-300A column (GRACE, USA) by high-performance liquid chromatography (HPLC). The mobile phase was composed of water, acetonitrile, and trifluoroacetic acid (proportions = 78%:22%:0.1%). The AE was collected by detection at an absorption value of 340 nm. Then, the isolated AE was mixed with hIMB1636-LDP protein at a molecular ratio of 1:3 and incubated at 4 °C overnight by gentle shaking to form the enediyne-integrated ADC hIMB1636-LDP-AE. Next, free AE (uncoupled) was removed by ultrafiltration centrifugation at 4000 rpm at 4 °C. The composition of hIMB1636-LDP-AE was finally confirmed by size-exclusion chromatography using a Delta Pak C4-300A column and Biosep™ SEC-s2000 column (Agela & Phenomenex, Washington, USA).

### Cell viability assessment with CCK-8

The cytotoxicity of hIMB1636-LDP-AE to Trop2-positive tumor cells was analyzed using Cell Counting Kit-8 (CCK-8). In brief, HCC827, H1975, MDA-MB-468, and MCF-7 cells were seeded in 96-well plates (5 × 10^3^ cells/well) and incubated for 24 h. The cells were then treated with different concentrations (0, 0.01, 0.03, 0.1, 0.3, 1, and 3 nM) of hIMB1636-LDP-AE, hIMB1636-LDP, or LDM for 48 h. After discarding the original culture medium, a mixture of 10 µL of CCK-8 reaction solution and 90 µL of complete culture medium was added to the wells and incubated for 1 h. The absorbance was measured at 450 nm using a microplate reader.

### Cell proliferation and migration assays using an xCELLigence RTCA system

For cellular proliferation, 50 µL of cell complete culture medium was added to each well of the RTCA E-16 plates, and base-line impedance was measured to ensure that all connections were in good condition. The HCC827, H1975, MDA-MB-468, or MCF-7 cells were harvested, counted, and then re-suspended in a culture medium. A volume of 100 µL cell suspension (5 × 10^4^ cells/mL) was added to each well of the E-16 plates and the cells were incubated at room temperature for 30 min to allow cell attachment. The cells were treated with PBS (as control), 1 nM, or 0.1 nM hIMB1636-LDP-AE. Finally, the plates were placed in the xCELLigence RTCA system (ACEA Biosciences Inc., California, USA) to record the cell index (CI) and monitored in real-time for 72 h.

Cell-migration experiments were performed using a CIM-Plate containing 16 wells, each of which was divided into upper and lower chambers. First, 165 µL of cell complete culture medium (10% FBS) and 30 µL of serum-free medium were added to the lower and upper chambers, respectively. Then the plates were installed in the xCELLigence instrument to measure background impedance. Cells were harvested and counted, and 5 × 10^4^ cells were re-suspended in 100 µL of serum-free medium and added to the upper chamber. The cells were subsequently treated with PBS (as control), 1 nM, or 0.1 nM hIMB1636-LDP-AE, and the plates were assembled onto the xCELLigence RTCA system for monitoring for 25 h or 42 h in real time.

### Bystander killing assay

The bystander-killing effect of hIMB1636-LDP-AE in a co-culture system containing H460 cells (low Trop2 expression) and HCC827 or MDA-MB468 cells (high Trop2 expression) was assessed by the RTCA system. The H460 cells were seeded in the E-Plate 16 at a density of 2500 cells/well. Then HCC827 or MDA-MB468 cells at 2500 cells/well were added to the wells, making the final ratio of 1:0, 0:1, or 1:1 of H460 to HCC827 or MDA-MB468 cells. Moreover, the cells were treated with 1 nM hIMB1636-MMAE. The cell growth curve was examined with the RTCA xCELLigene system.

### Analysis of cancer stem cell (CSC) marker expression

Flow cytometry was used to analyze the effects of hIMB1636-LDP-AE on a variety of CSCs. Trop2-positive tumor cells were digested with trypsin, seeded in six-well plates, and cultured for 24 h. Subsequently, the cells were treated with 1 nM hIMB1636-LDP-AE for 24 h. The cells were collected, washed, and incubated for 2 h with fluorochrome-conjugated antibodies (anti-CD44 was conjugated with PE (BioLegend, 397503, 1:100), and anti-CD24 was conjugated with APC (BioLegend, 311117, 1:100)). Finally, cells were washed with cold PBS and analyzed by flow cytometry. The data were analyzed using ACEA NovoExpress software. The FACS gating strategies were provided in Supplementary Fig. S5. Then, the protein levels of CSC markers were assessed by the previously mentioned protein extraction and western-blot analysis. Antibodies against OCT4 (Proteintech, 11263-1-AP, 1:1000), EpCAM (Proteintech, 21050-1-AP, 1:1000), SOX2 (Proteintech, 66411-1-Ig, 1:1000) and Nanog (Cell Signaling Technology, 4903 T, 1:1000) were used. Uncropped scans of western blots were provided in Supplementary Fig. S3.

### Tumorsphere formation assay

HCC827, H1975, MDA-MB-468, or MCF-7 cells were digested with trypsin, counted, and resuspended in complete StemXVivo Tumor Sphere Media. Then, 100 µL of cells in suspension were seeded into a special 96-well plate (3000 cells/well) and treated with different concentrations of hIMB1636-LDP-AE (0, 0.5, and 1 nM). The cells were cultured in an incubator in an atmosphere with 5% CO_2_ at 37 °C, and tumorspheres were counted and photographed after 10 days of incubation.

### Analyses of apoptosis and cell-cycle arrest

Cells (5 × 10^4^ cells/well) were incubated in a six-well plate for 24 h and then treated with 1, 10, or 50 nM hIMB1636-LDP-AE for 24 h, and the untreated cells served as controls. Next, all cells were harvested with trypsin, and washed with PBS. For apoptosis analysis, 2 × 10^5^ cells were resuspended in 500 μL of binding buffer and stained with 5 μL of annexin-V FITC and 10 μL propidium iodide (PI) for 15 min at room temperature in the dark. The stained cells were detected and analyzed using an ACEA NovoExpress flow cytometer within 1 h. For cell-cycle arrest analysis, 5 × 10^5^ cells were treated as described above and collected. Next, 1 mL of DNA staining solution and 10 μL of permeabilization solution were added per cell sample and incubated for 0.5 h at room temperature in the dark. Finally, all cell samples were analyzed and cycle distribution was calculated using ACEA NovoExpress software. The FACS gating strategies are given in Supplementary Fig. S6.

### Antitumor efficacy of hIMB1636-LDP-AE in vivo

For in vivo experiments, 6-week-old female BALB/c nude mice were purchased from Beijing HFK Bioscience Co., Ltd. [Beijing, China, SCXK(Beijing)2019-0008]. The animals were maintained in animal facilities at the Institute of Medicinal Biotechnology under Specific Pathogen-Free (SPF) conditions. All animal studies were approved by the Ethics Committee of the Institute of Medicinal Biotechnology, Chinese Academy of Medical Sciences. The animal experiments were performed in accordance with ARRIVE guidelines^52^.

For the HCC827 tumor xenograft model, 5.0 × 10^6^ cells in 100 μL PBS were injected subcutaneously into the right flank of each BALB/c nude mouse. When tumor volumes reached approximately 80 mm^3^, the mice were randomly allocated to six groups (*n* = 6 per group) and injected intravenously with 0.8 mg/kg hIMB1636, 0.045 mg/kg LDM, or different concentrations of hIMB1636-LDP-AE every five days for a total of four injections; the control group received only PBS. We monitored tumor growth every four days by measuring tumor length (L) and width (W) using Vernier calipers and measured the body weights of all mice. Tumor volume (TV) was calculated according to the following formula: TV = 0.5 × L × W^2^. The mice were euthanized via CO_2_ asphyxiation at the end of the experiment, and other organs including the heart, liver, spleen, and lung were harvested and fixed in 4% paraformaldehyde for hematoxylin-eosin (H&E) staining.

For the construction of H1975, MDA-MB-468, and MCF-7 xenograft model, cells (5.0 × 10^6^ in 100 μL of 70 μL 1 × PBS and 30 μL BD Matrigel Matrix) were injected subcutaneously into the right flank of each BALB/c nude mouse. When tumor volumes reached approximately 60-80 mm^3^, the mice were randomly allocated to two groups (*n* = 6 per group) and injected intravenously with 0.8 mg/kg hIMB1636-LDP-AE every five days for a total of four injections; the control group received only PBS. Then the tumor growth and body weights were monitored as described above. For the detection of hematological parameters, the mice were anesthetized with isoflurane, and then blood samples from different groups were collected in EDTA-K2 containing anticoagulant tubes by retro-orbital bleeding. The following parameters were measured by HITACHI7100 Automatic Aralyzer (Japan): Hemoglobine (g/L), Erythrocytes (*10^12^/L), Leukocytes (*10^9^/L), Neutrophils (*10^9^/L), and Platelet (*10^9^/L). Finally, the mice were euthanized via CO_2_ asphyxiation at the end of the experiment and tumors were harvested and weighed.

### Side population (SP) analysis

For SP cell analysis, the HCC827 tumor tissue treated with different compounds was minced, ground, and then filtered to obtain a tumor single-cell suspension. Then tumor cells were washed and resuspended in pre-warmed (37 °C) cell culture medium at a concentration of 1 × 10^6^ cells/ml. Cells from the same sample were divided into experimental and control groups. The experimental cells were directly added DCV for staining; The control group was added verapamil (blocking cell membrane ion channels), incubated at 37 °C for 15 min, and then added DCV for staining. Cells were incubated for 90 min at 37 °C with gentle vortexing every 15 min. Thereafter, extracellular DCV was washed away in 5× volume ice-cold PBS. Cells were stained with PI strictly according to the manufacturer’s instructions. Cells were kept on ice and immediately analyzed on a flow cytometer. DCV blue and red fluorescence signals excited by the violet laser were detected using a 450/50 nm and a 585/42 nm bandpass filter, respectively. The FACS gating strategies are given in Supplementary Fig. S7.

### Statistical analysis

We conducted data analysis using GraphPad Prism 7 software, and the results were presented as means ± standard deviation (SD). Statistical significance among groups was determined by a one or two-way ANOVA followed by Tukey’s test, and the statistical significance between two groups was analyzed by applying the unpaired Student’s *t* test. IC_50_ values were determined by nonlinear regression analysis of concentration-response curves using SPSS 17.0. All experiments were repeated more than three times, *P* < 0.05 was considered statistically significant.

### Reporting summary

Further information on research design is available in the Nature Research Reporting Summary linked to this article.

### Supplementary information

Supplementary Figures

REPORTING SUMMARY

## Data Availability

The data generated in this study are included in the main article and its supplementary data. All reasonable requests for resources and reagents should be directed and will be fulfilled by the corresponding author. The materials and data will be made available upon request after the completion of a material transfer agreement. The authors declare that all data supporting the findings of this study are available within the paper.
