# Supplementary material for: A new TROP2-targeting antibody-drug conjugate shows potent antitumor efficacy in breast and lung cancers
Source: NPJ Precis Oncol. 2024 Apr 23;8:94. doi: 10.1038/s41698-024-00584-z (PMC11039471; doi:10.1038/s41698-024-00584-z)
Supplement: Supplementary file 1 — Supplementary Figures [file 41698_2024_584_MOESM1_ESM.pdf]

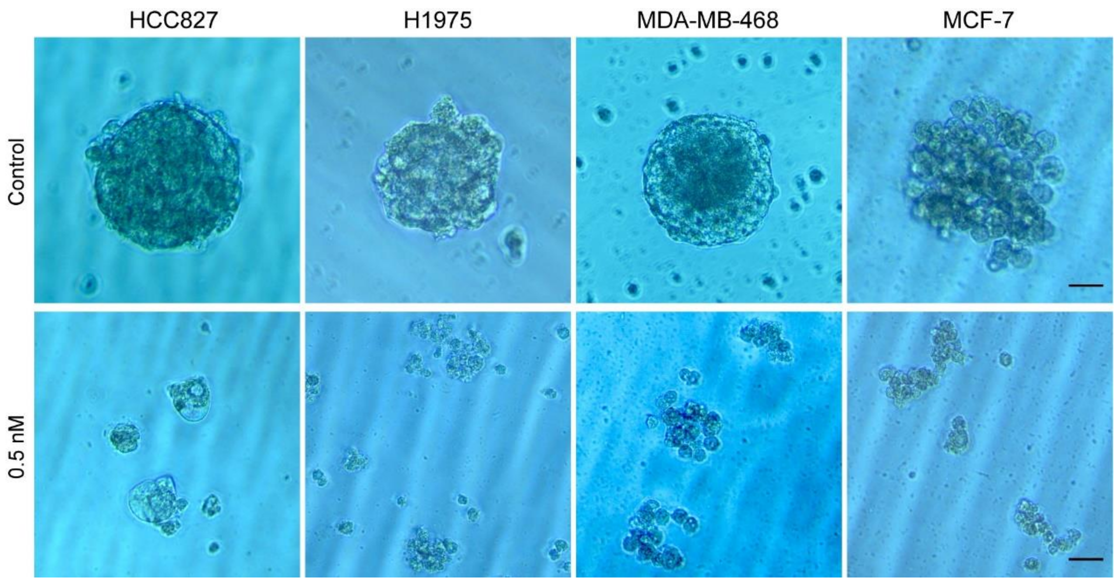

Supplementary Fig. S1 Representative graph of CSC self-renewal capacity in secondary spheres of primary cancer cells. Scale bars in (**S1**) represent 30 μm.

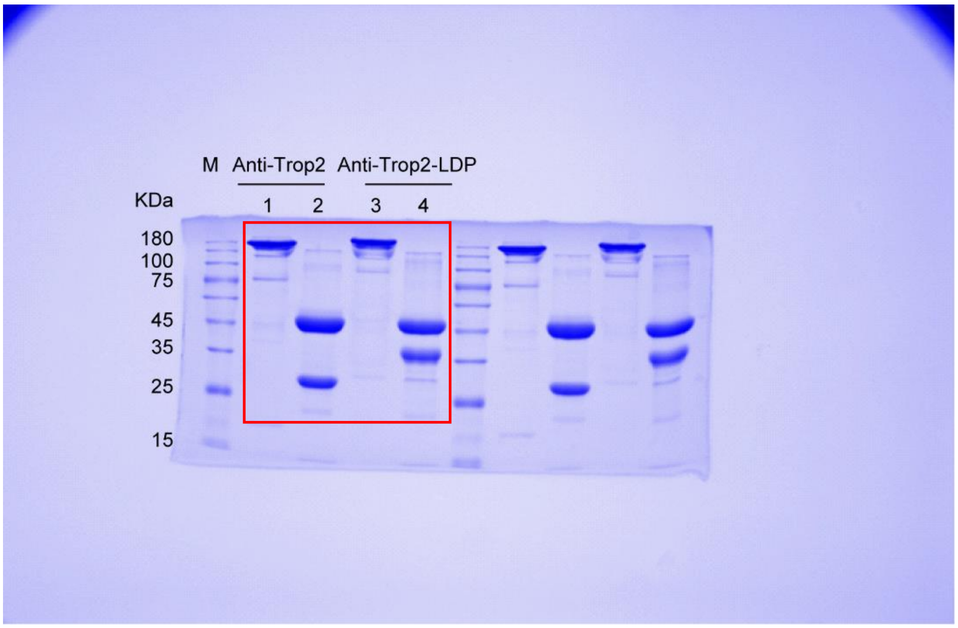

Supplementary Fig. S2. Uncropped scans of the gel in Fig 1b. The red border is the gel region shown in article.

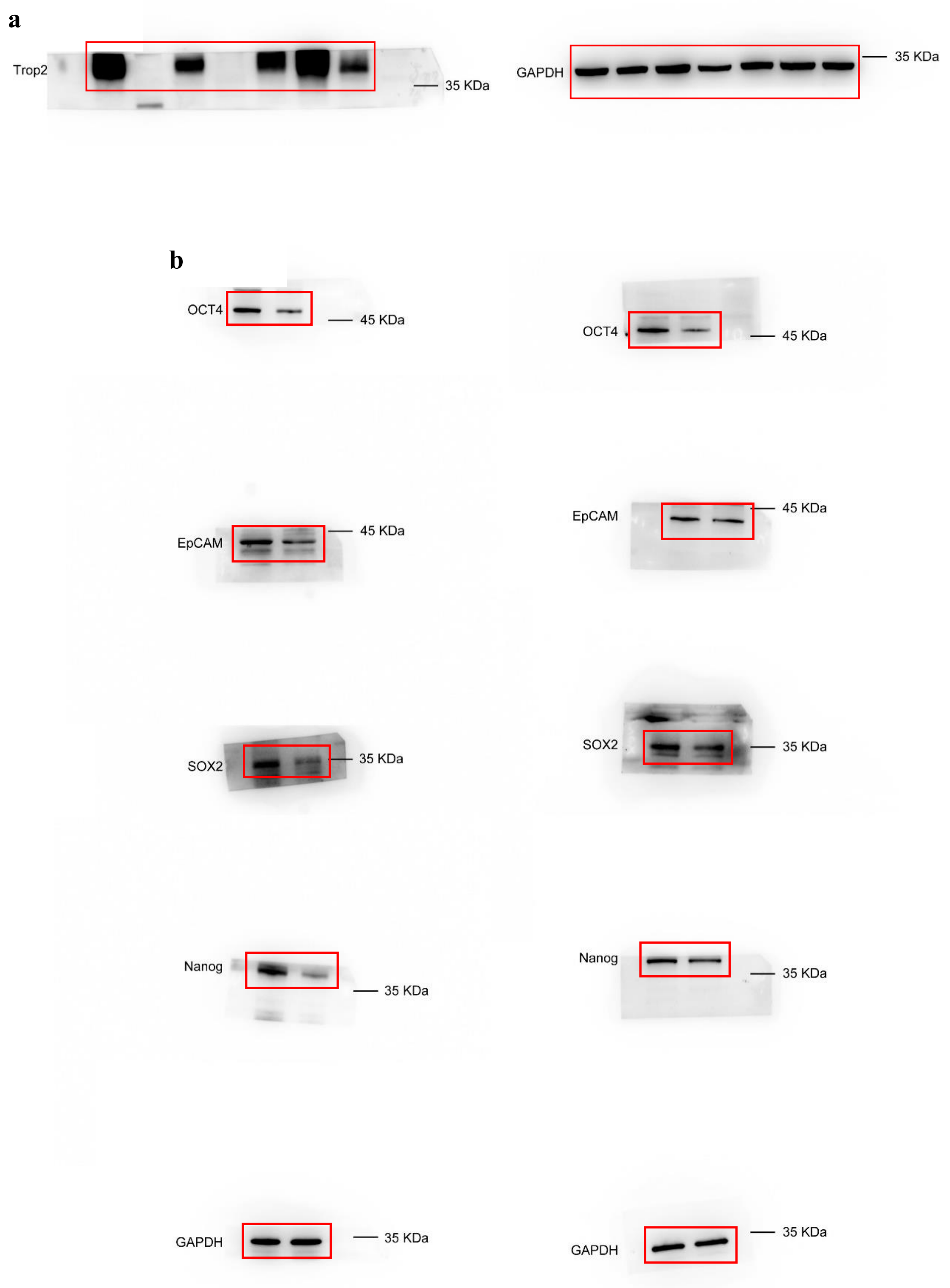
a
Trop2
35 KDa
GAPDH
35 KDa
b
OCT4
45 KDa
OCT4
45 KDa
EpCAM
45 KDa
EpCAM
45 KDa
SOX2
35 KDa
SOX2
35 KDa
Nanog
35 KDa
Nanog
35 KDa
GAPDH
35 KDa
GAPDH
35 KDa

c

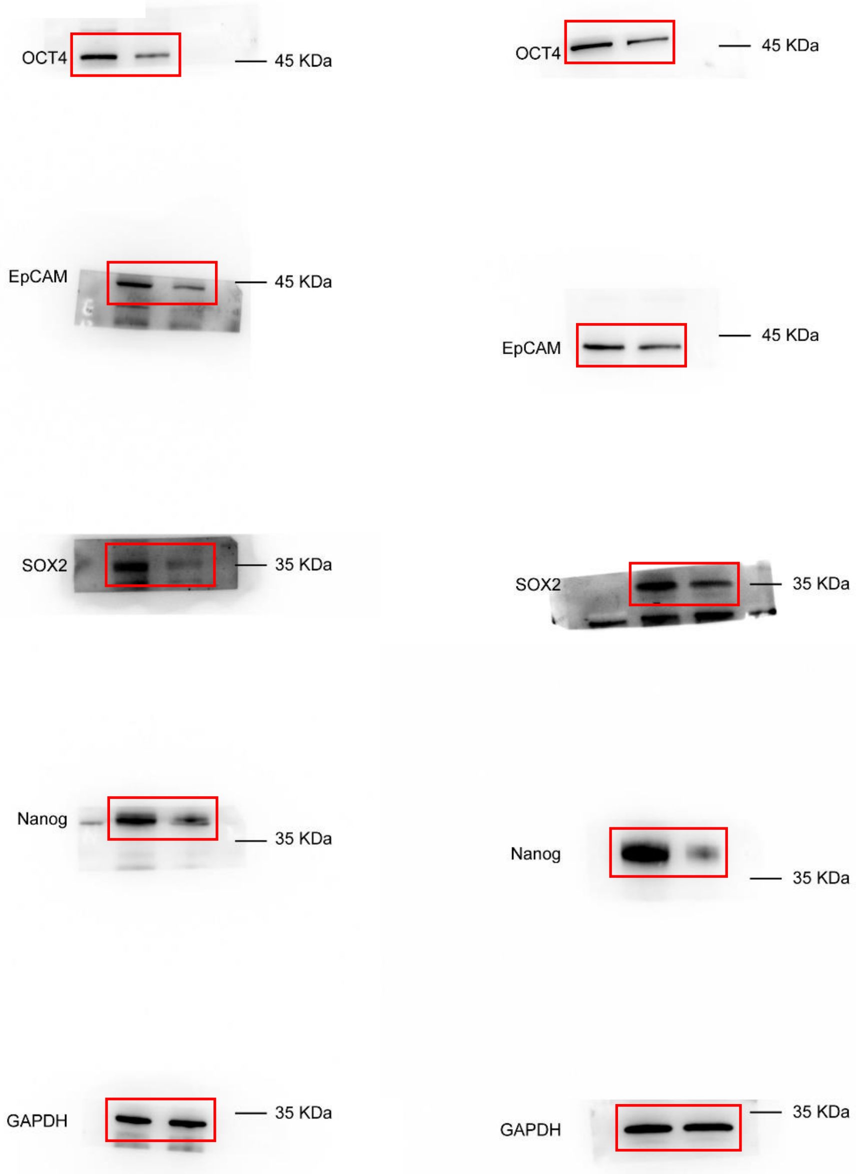

Supplementary Fig. S3. Uncropped gel images for western blots. Red boxes indicate gel regions shown in article. (**a**) Corresponds to Fig.1f. (**b**) Corresponds to Fig.5d. (**c**) Corresponds to Fig.5e.

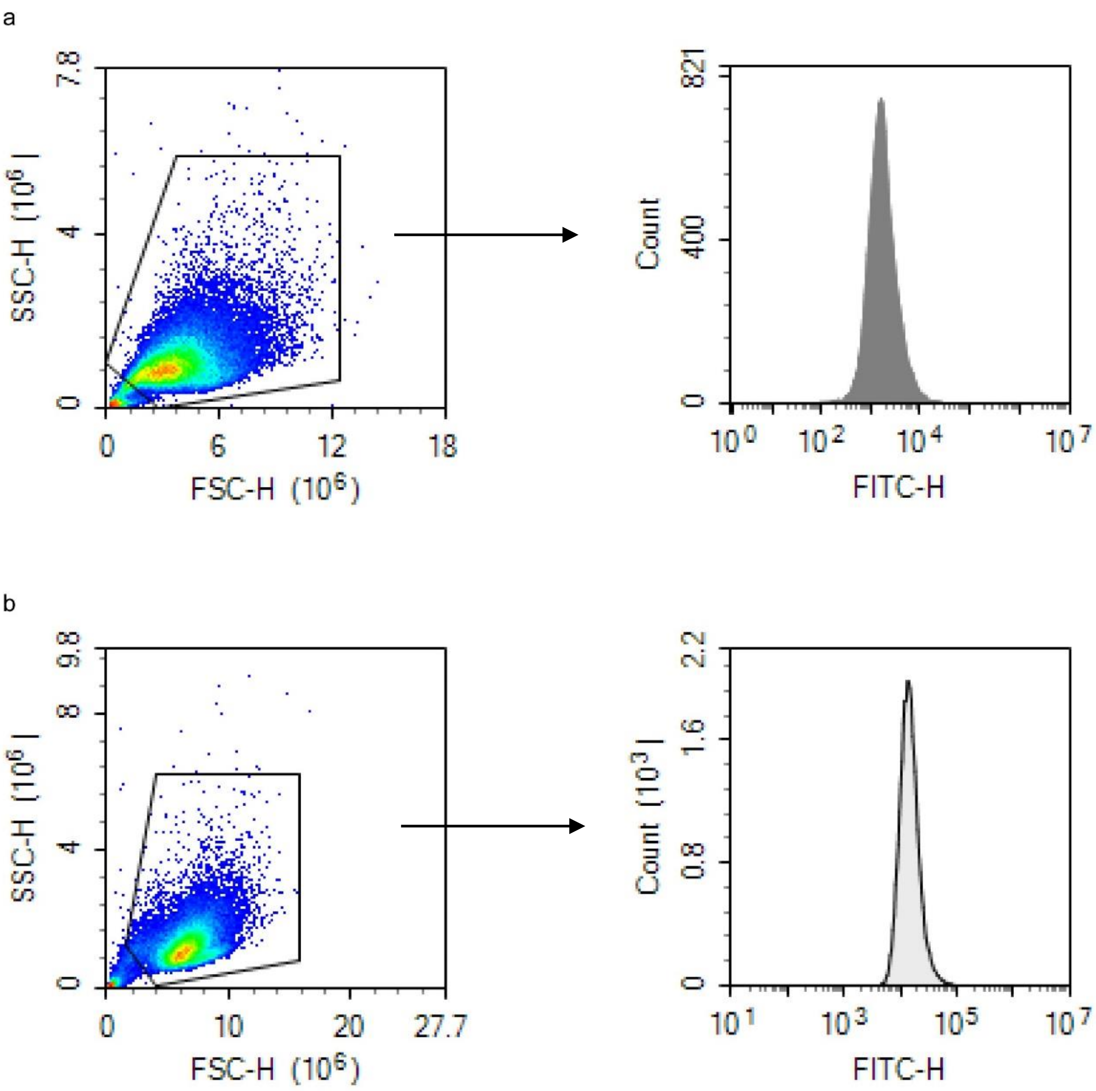

Supplementary Fig. S4 Gating strategies for binding of related antibodies to Trop2 antigens on the cell surface. (**a**) Gating strategy for binding activity of hIMB1636-LDP antibody to different tumor cell lines shown in Fig. 1g and Fig. 3e. (**b**) Gating strategy for the specific binding of hIMB1636-LDP and hIMB1636 to natural antigen on tumor cells shown in Fig. 2a-c and Fig. 5h.

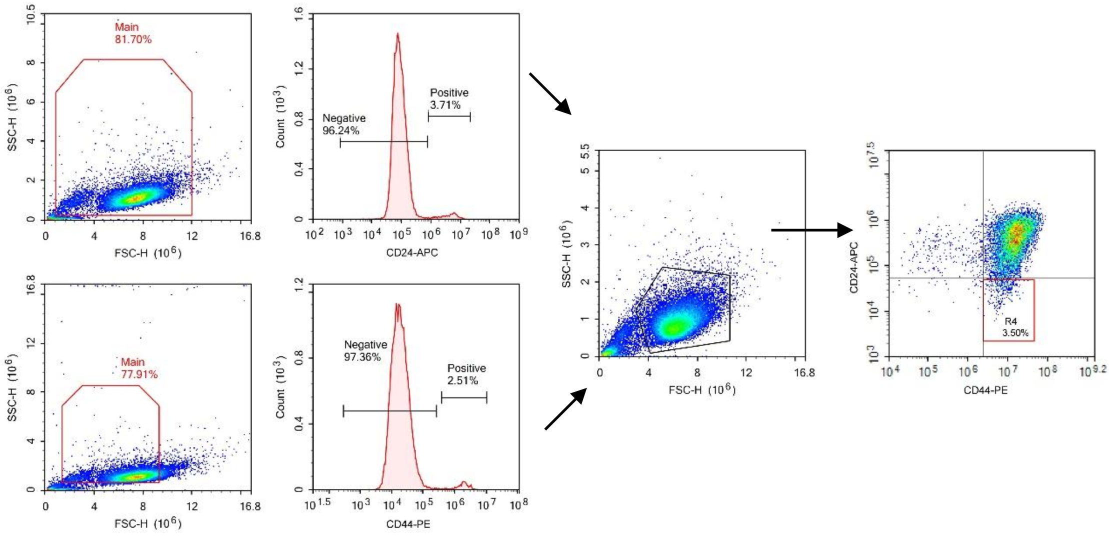

Supplementary Fig. S5 Gating strategies used for cancer stem cells sorting shown in Fig. 5a-b.

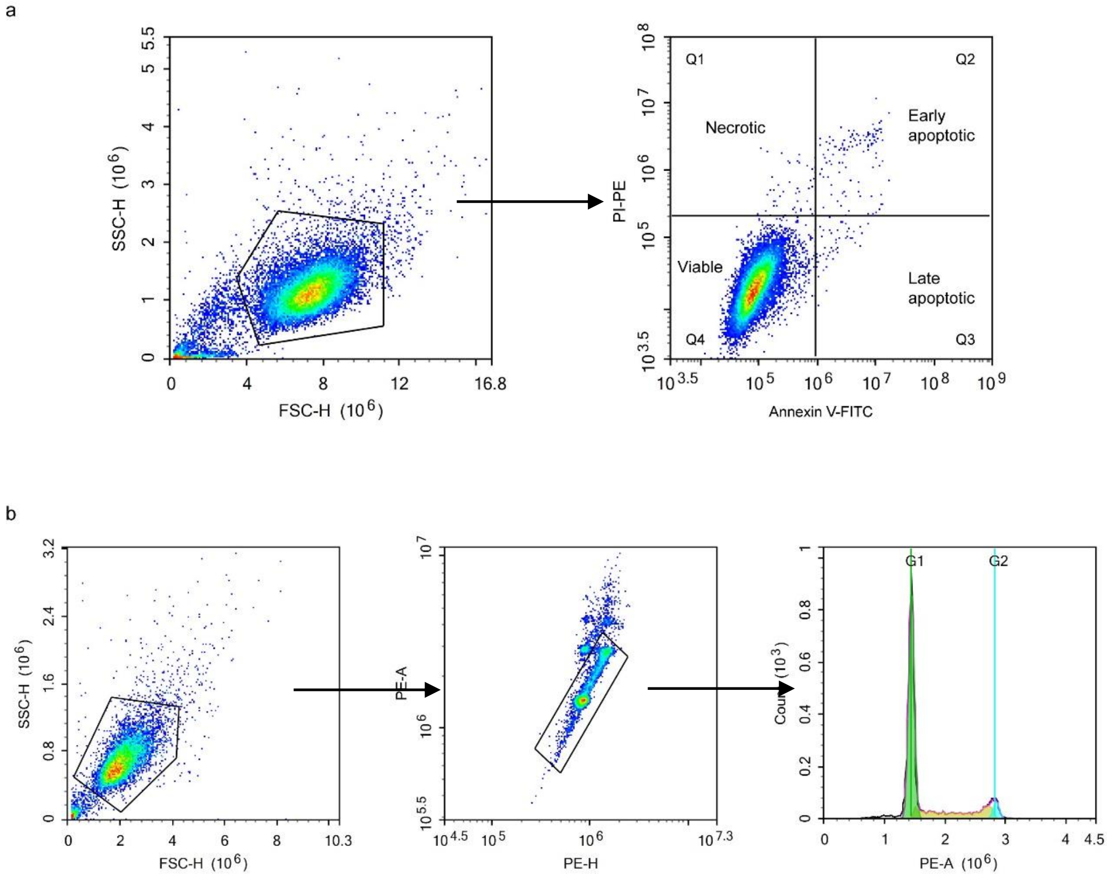

Supplementary Fig. S6 Gating strategies for apoptosis and cell-cycle arrest in cancer cells after hIMB1636-LDP-AE treatment. (**a**) Gating for sorting propidium iodide (PI)/annexin V-stained different tumor cell lines shown in Fig. 6a-b. The

different quadrants represent distinct apoptotic statuses, categorized as follows: Q1 (necrotic), Q2 (late apoptotic), Q3 (early apoptotic), and Q4 (viable, non-apoptotic). (**b**) Gating strategy for hIMB1636-LDP-AE induced cell-cycle arrest shown in Fig. 6c-f.

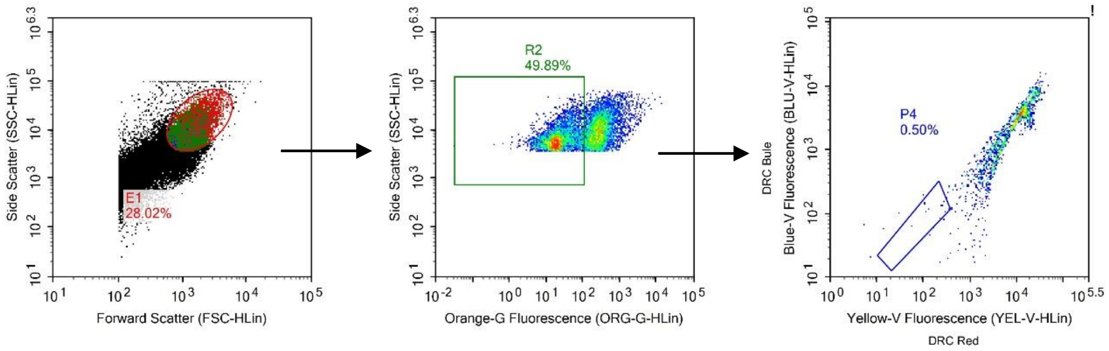

Supplementary Fig. S7 Gating strategy for sorting side population cells from tumor tissues shown in Fig. 8e.
