# Supplementary material for: A new TROP2-targeting antibody-drug conjugate shows potent antitumor efficacy in breast and lung cancers
Source: NPJ Precis Oncol. 2024 Apr 23;8:94. doi: 10.1038/s41698-024-00584-z (PMC11039471; doi:10.1038/s41698-024-00584-z)
Supplement: Supplementary file 2 — REPORTING SUMMARY [file 41698_2024_584_MOESM2_ESM.pdf]

nature portfolio

Corresponding author(s): DAPR NPJPRECISIONONCOLOGY-04043R

Last updated by author(s): Feb 13, 2024

Reporting Summary

Nature Portfolio wishes to improve the reproducibility of the work that we publish. This form provides structure for consistency and transparency in reporting. For further information on Nature Portfolio policies, see our Editorial Policies and the Editorial Policy Checklist.

Statistics

For all statistical analyses, confirm that the following items are present in the figure legend, table legend, main text, or Methods section.

n/a	Confirmed	
☐	☒	The exact sample size (*n*) for each experimental group/condition, given as a discrete number and unit of measurement
☐	☒	A statement on whether measurements were taken from distinct samples or whether the same sample was measured repeatedly
☐	☒	The statistical test(s) used AND whether they are one- or two-sided<br>*Only common tests should be described solely by name; describe more complex techniques in the Methods section.*
☒	☐	A description of all covariates tested
☒	☐	A description of any assumptions or corrections, such as tests of normality and adjustment for multiple comparisons
☐	☒	A full description of the statistical parameters including central tendency (e.g. means) or other basic estimates (e.g. regression coefficient) AND variation (e.g. standard deviation) or associated estimates of uncertainty (e.g. confidence intervals)
☐	☒	For null hypothesis testing, the test statistic (e.g. F, t, r) with confidence intervals, effect sizes, degrees of freedom and P value noted<br>*Give P values as exact values whenever suitable.*
☒	☐	For Bayesian analysis, information on the choice of priors and Markov chain Monte Carlo settings
☒	☐	For hierarchical and complex designs, identification of the appropriate level for tests and full reporting of outcomes
☐	☒	Estimates of effect sizes (e.g. Cohen's *d*, Pearson's *r*), indicating how they were calculated

Our web collection on statistics for biologists contains articles on many of the points above.

Software and code

Policy information about availability of computer code

Data collection	Experimental data obtained through different experimental operation.
Data analysis	GraphPad Prism 7 software,SPSS 17.0, Biacore T200 evaluation software, xCELLigence RTCA system, a Olympus Microsystems confocal fluorescence microscope, a Xenogen IVIS-200 system, ACEA NovoExpress software and so on.

For manuscripts utilizing custom algorithms or software that are central to the research but not yet described in published literature, software must be made available to editors and reviewers. We strongly encourage code deposition in a community repository (e.g. GitHub). See the Nature Portfolio guidelines for submitting code & software for further information.

Data

Policy information about availability of data

All manuscripts must include a data availability statement. This statement should provide the following information, where applicable:

- Accession codes, unique identifiers, or web links for publicly available datasets
- A description of any restrictions on data availability
- For clinical datasets or third party data, please ensure that the statement adheres to our policy

Data available on request from the authors.

Research involving human participants, their data, or biological material

Policy information about studies with human participants or human data. See also policy information about sex, gender (identity/presentation), and sexual orientation and race, ethnicity and racism.

Reporting on sex and gender	Not involved
Reporting on race, ethnicity, or other socially relevant groupings	Not involved
Population characteristics	Not involved
Recruitment	Not involved
Ethics oversight	All animal studies were approved by the Ethics Committee of the Institute of Medicinal Biotechnology, Chinese Academy of Medical Sciences. The animal experiments were performed in accordance with ARRIVE guidelines.

Note that full information on the approval of the study protocol must also be provided in the manuscript.

Field-specific reporting

Please select the one below that is the best fit for your research. If you are not sure, read the appropriate sections before making your selection.

☒ Life sciences ☐ Behavioural & social sciences ☐ Ecological, evolutionary & environmental sciences

For a reference copy of the document with all sections, see nature.com/documents/nr-reporting-summary-flat.pdf

Life sciences study design

All studies must disclose on these points even when the disclosure is negative.

Sample size	All experiments were repeated more than three times. For in vivo experiments, the mice were randomly allocated to different groups (n = 6 per group).
Data exclusions	Data were not excluded from analysis. All experiments were repeated more than three times, and selected typical diagram.
Replication	All replication attempts were successful, and the experiments were repeated more than three times.
Randomization	For in vivo experiments, the mice were randomly allocated to different groups. Other experiments were analyzed equally with no sub-sampling and thus, there was no requirement for randomization.
Blinding	Blinding was not possible as experimental conditions were evident from the image data. All experimental results are analyzed from objective data, and there is no subjective consciousness.

Reporting for specific materials, systems and methods

We require information from authors about some types of materials, experimental systems and methods used in many studies. Here, indicate whether each material, system or method listed is relevant to your study. If you are not sure if a list item applies to your research, read the appropriate section before selecting a response.

Materials & experimental systems

n/a	Involved in the study	
☐	☒	Antibodies
☐	☒	Eukaryotic cell lines
☒	☐	Palaeontology and archaeology
☐	☒	Animals and other organisms
☒	☐	Clinical data
☒	☐	Dual use research of concern
☒	☐	Plants

Methods

n/a	Involved in the study	
☒	☐	ChIP-seq
☐	☒	Flow cytometry
☒	☐	MRI-based neuroimaging

Antibodies

Antibodies used	Primary antibodies against LAMP1 (9091S), and Nanog (4903T), anti-OCT4 (11263-1-AP), anti-EpCAM (21050-1-AP), anti-SOX2

Antibodies used	(66411-1-Ig), anti-Trop2 antibody (10428-MM02) and anti-GAPDH antibody, used at 1:1000. The secondary antibodies conjugated to FITC or HRP, used at 1:200. Anti-CD24 antibody conjugated with APC (311117) and anti-CD44 antibody conjugated with PE (397503), used at 1:100.
Validation	Primary antibodies against LAMP1 (9091S), and Nanog (4903T) were purchased from Cell Signaling Technology (Boston, USA). The antibodies anti-OCT4 (11263-1-AP), anti-EpCAM (21050-1-AP), and anti-SOX2 (66411-1-Ig) were obtained from Proteintech Group, Inc. (Chicago, USA). Anti-Trop2 antibody (10428-MM02) was purchased from Sino Biological (Guangzhou, China). A primary antibody for GAPDH, and the secondary antibodies conjugated to FITC or HRP were obtained from ZSGB-Bio (Beijing, China). Anti-CD24 antibody conjugated with APC (311117) and anti-CD44 antibody conjugated with PE (397503) were purchased from BioLegend (Beijing, China). All antibodies have been validated in other studies (see website).

Eukaryotic cell lines

Policy information about cell lines and Sex and Gender in Research

Cell line source(s)	A lung cancer cell line (HCC827) and breast cancer cell lines (MDA-MB-468, MCF-7) were obtained from the Cell Center of Peking Union Medical College (Beijing, China). Lung cancer cell lines (H1975, A549, H460) and breast cancer cell line MDA-MB-231 were preserved in our laboratory.
Authentication	Although none of these cell lines have been authenticated, we are confident that they are correct.
Mycoplasma contamination	Cell lines were not tested for mycoplasma contamination but no indication of contamination was observed.
Commonly misidentified lines (See ICLAC register)	No commonly misidentified cell lines were used.

Animals and other research organisms

Policy information about studies involving animals; ARRIVE guidelines recommended for reporting animal research, and Sex and Gender in Research

Laboratory animals	6-week-old female BALB/c nude mice were purchased from Beijing HFK Bioscience Co., Ltd. [Beijing, China, SCXK(Beijing)2019-0008]. The animals were maintained in animal facilities at the Institute of Medicinal Biotechnology under Specific Pathogen-Free (SPF) conditions.
Wild animals	No wild animals were used in this study.
Reporting on sex	Not involved.
Field-collected samples	No field-collected samples were used in this study.
Ethics oversight	All animal studies were approved by the Ethics Committee of the Institute of Medicinal Biotechnology, Chinese Academy of Medical Sciences. The animal experiments were performed in accordance with ARRIVE guidelines.

Note that full information on the approval of the study protocol must also be provided in the manuscript.

Plants

Seed stocks	*Report on the source of all seed stocks or other plant material used. If applicable, state the seed stock centre and catalogue number. If plant specimens were collected from the field, describe the collection location, date and sampling procedures.*
Novel plant genotypes	*Describe the methods by which all novel plant genotypes were produced. This includes those generated by transgenic approaches, gene editing, chemical/radiation-based mutagenesis and hybridization. For transgenic lines, describe the transformation method, the number of independent lines analyzed and the generation upon which experiments were performed. For gene-edited lines, describe the editor used, the endogenous sequence targeted for editing, the targeting guide RNA sequence (if applicable) and how the editor was applied.*
Authentication	*Describe any authentication procedures for each seed stock used or novel genotype generated. Describe any experiments used to assess the effect of a mutation and, where applicable, how potential secondary effects (e.g. second site T-DNA insertions, mosiacism, off-target gene editing) were examined.*

Flow Cytometry

Plots

Confirm that:

☒ The axis labels state the marker and fluorochrome used (e.g. CD4-FITC).

☒ The axis scales are clearly visible. Include numbers along axes only for bottom left plot of group (a 'group' is an analysis of identical markers).

☒ All plots are contour plots with outliers or pseudocolor plots.

☒ A numerical value for number of cells or percentage (with statistics) is provided.

Methodology

Sample preparation	The cell samples was collected from different tumor cell lines. Positive cell clones were expanded cultures for hIMB1636 antibody preparation, then cell culture medium was collected and the recombinant protein hIMB1636-LDP and hIMB1636 antibody were purified by HitrapTM protein G columns (GE Healthcare, Chicago, USA) according to the manufacturer's instructions. For tumor tissue samples, the mice were euthanized at the end of the experiment, and tumor were harvested and the tumor single cell suspensions were prepared according to experimental procedures.
Instrument	ACEA Biosciences Inc., California, USA
Software	ACEA NovoExpress software
Cell population abundance	Not involved.
Gating strategy	The gate is selected objectively according to different experimental requirements.

☐ Tick this box to confirm that a figure exemplifying the gating strategy is provided in the Supplementary Information.
